# Building a cluster of NLR genes conferring resistance to pests and pathogens: the story of the *Vat* gene cluster in cucurbits

**DOI:** 10.1038/s41438-021-00507-0

**Published:** 2021-04-01

**Authors:** Véronique Chovelon, Rafael Feriche-Linares, Guillaume Barreau, Joël Chadoeuf, Caroline Callot, Véronique Gautier, Marie-Christine Le Paslier, Aurélie Berad, Patricia Faivre-Rampant, Jacques Lagnel, Nathalie Boissot

**Affiliations:** 1 INRAE, GAFL, 84143 Montfavet, France; 2 INRAE, CNRGV, 31326 Castanet-Tolosan, France; 3 INRAE, GDEC-Gentyane Plateform, 63000 Clermont-Ferrand, France; 4 Université Paris-Saclay, INRAE, EPGV, 91000 Evry-Courcouronnes, France

**Keywords:** Biological sciences, Plant sciences

## Abstract

Most molecularly characterized plant resistance genes (R genes) belong to the nucleotide-binding-site-leucine-rich-repeat (NLR) receptor family and are prone to duplication and transposition with high sequence diversity. In this family, the *Vat* gene in melon is one of the few R genes known for conferring resistance to insect, i.e., *Aphis gossypii*, but it has been misassembled and/or mispredicted in the whole genomes of Cucurbits. We examined 14 genomic regions (about 400 kb) derived from long-read assemblies spanning *Vat*-related genes in *Cucumis melo*, *Cucumis sativus, Citrullus lanatus, Benincasa hispida, Cucurbita argyrosperma*, and *Momordica charantia*. We built the phylogeny of those genes. Investigating the paleohistory of the *Vat* gene cluster, we revealed a step by step process beginning from a common ancestry in cucurbits older than 50 my. We highlighted *Vat* exclusively in the *Cucumis* genera, which diverged about 20 my ago. We then focused on melon, evaluating a minimum duplication rate of *Vat* in 80 wild and cultivated melon lines using generalist primers; our results suggested that duplication started before melon domestication. The phylogeny of 44 Vat-CDS obtained from 21 melon lines revealed gain and loss of leucine-rich-repeat domains along diversification. Altogether, we revealed the high putative recognition scale offered in melon based on a combination of SNPs, number of leucine-rich-repeat domains within each homolog and number of homologs within each cluster that might jointly confer resistance to a large pest and pathogen spectrum. Based on our findings, we propose possible avenues for breeding programs.

## Introduction

Most molecularly characterized plant resistance genes (R genes) belong to the nucleotide-binding-site-leucine-rich-repeat (NLR) receptor family and are prone to duplication and transposition with high sequence diversity. Consequently, in eudicots, for instance, on average 63% of these genes clustered^1^, with diversity observed in terms of so-called copy number variation (CNV). This diversity within R gene clusters among crops and their wild relatives could be a major source of resistance to diseases and pests, but their introgression in cultivars is still hindered by several factors. First, there are only a few studied cases of diversity in the number and organization of units within R gene clusters (mostly in rice and *Arabidopsis*) given that sequencing/assembling these genomic regions with short-read is challenging. Second, it is tricky to distinguish specific sequences responsible for the resistance phenotype from homologous genes. Hence, building specific molecular markers for different NLRs in complex clusters has been out of reach and would be urgently needed to enhance most plant breeding programs.

Plant resistance to insects has been particularly poorly characterized in this context with only four known NLR genes targeting insects^2^. Remarkably, all of these insects—aphids, whiteflies, and psyllids—belong to the Hemipteran family of insects that furtively puncture and inject saliva into mesophyll cells. This saliva contains effectors that could be putatively recognized by some NLR proteins that are considered to be immune sensors^3^. In this study, we focused on an NLR gene cluster containing the *Vat* NLR gene, conferring resistance to the melon aphid (*Aphis gossypii*) in *Cucumis melo*^4^. Cisgenesis with *Vat-1*, isolated from the melon line PI 161375, showed it controlled the resistance to *A. gossypii* and to potyviruses when plants were inoculated by this aphid species^4^. Actually, this resistance to virus is aphid clone dependent^5^ and genetic studies suggested that tightly linked genes are involved. The genomic region spanning these genes is now called the Vat cluster^6^.

*Vat-1* (five exons and four introns), encoded a predicted 1467-amino acid protein belonging to the coiled-coil (CC)-NLR family^4^. The NB domain is suggested to act as a molecular switch of the resistance response, and the LR part is believed to be the initial recognition domain of the pathogen^7^. In the LR part, SNP and length polymorphism were observed^4,8^. Length polymorphism occurred in a specific domain, called D or LRR2. This domain was found to consist of near-perfect repeats of 65 amino acids, which we called R65aa, while *Vat* homologs containing two to five R65aa have been identified. In PI 161375 line, a *Vat* homolog is located at 16,676 bp of *Vat-1*, suggesting tight clustering of the *Vat*-related sequences. Actually, 23 genes of the NLR family have been identified in a 1-Mb region containing *Vat*^9^, corresponding to the highest density of resistance genes in melon^10^ but genome assemblies from short reads have not been satisfying to decipher the Vat cluster^6^ and therefore diversity of the Vat cluster is still very partially known.

In this study, we investigated the Vat cluster history in the cucurbit family using genomes assembled from long reads which potentially encompass a complete *Vat* homolog or even several successive *Vat* homologs. Then we focused on understanding how *Vat* homolog diversity builds up studying a large set of melon lines.

## Results

### Architecture of the genomic region spanning *Vat* genes in cucurbits

We obtained 300–350 kb containing several *Vat* homologs in seven melon lines: PI 161375 (a Korean line), Anso77 (a Spanish line), Doublon (a French line), Payzawat and HS (both Chinese lines), DHL92 (a doubled haploid line derived from a cross between PI 161375 and the Spanish line Piel de Sapo), and Harukei-3 (a Japanese line). We first obtained a 610 kb contig for PI 161375 by assembling seven BACs sequenced using PacBio technology (Methods S1 and Fig. S1), one of which had previously been identified as spanning two *Vat* homologs^4^. From this sequence, we defined a 301.5 kb genomic region between M5 and M4 genetic makers containing all of the *Vat* homologs we identified within the 610 kb region. We used a blast/mapping strategy to retrieve sequences from M5 to M4 in assemblies of six other melon lines (Data S1). A 323.5 kb sequence was identified within a 6987 kb contig of Anso77 (Data S2), while a 347.5 kb sequence was identified within a 6871 kb contig of Doublon (Data S2), and both genomes were obtained by long-read assembly (Methods S1). For the four other lines we retrieved M5-M4 sequences of 337.6 and 376.7 kb (Data S2), from public databases. M5-M4 sequences in PI 161375, Anso77, Doublon, HS, and Harukei-3 were fully syntenic, with a subtelomeric region of chromosome 5 in DHL92 but with the opposite arm of chromosome 5 in Payzawat (Fig. S2), suggesting pericentric inversion between DHL92 and Payzawat lines. Then we checked the long-read coverage at the putative inversion breakpoints. For Payzawat, no long reads overlapped the breakpoints (along 300 bp) and conversely for DHL92 many long reads overlapped the breakpoints.

We investigated microsynteny within and between the seven M5-M4 regions. Dot plots highlighted several duplications within each M5-M4 region except DHL92 (Fig. S3-A) with small palindromic sequences in most of them, thus suggesting that repeat elements were present. We designed six markers in the M5-M4 region of PI 161375 (Fig. S1) and located them by alignment (blastn) in the seven melon lines (Data S1)—they mapped in the same order in all lines. Syntenic analyses carried with DHL92 as reference, actually revealed different indels between the M5-M4 regions (Fig. S3-B). All accessions shared large syntenic blocks downstream M5 (almost 60 kb) and upstream M4 (about 100 kb). The most compact M5-M4 region was in PI 161375, and remarkably large insertions occurred in HS, DHL92, and Anso77. DHL92 was derived from a cross between Piel de Sapo and PI 161375—because of the differences we observed in the M5-M4 region between DHL92 and PI 161375, we concluded that the M5-M4 region in DHL92 corresponded to the M5-M4 region in Piel de Sapo.

**Fig. 1:**
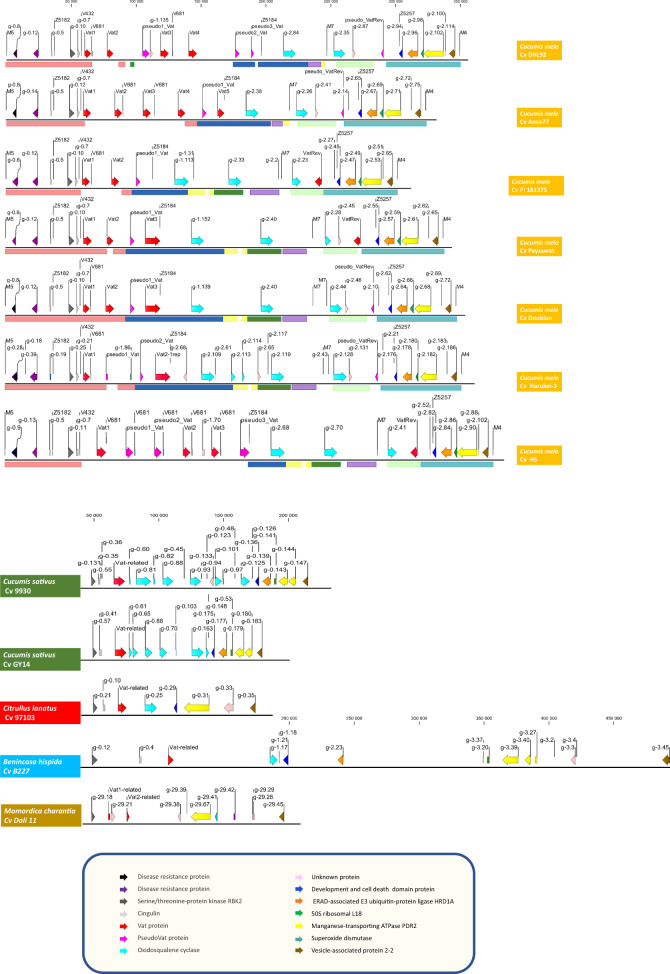
Genes identified after manual annotation for *Vat*-related sequences and MAKER annotation^45^ within M5-M4 regions. The sequences were obtained from public databases for *Cucumis melo* DHL92 (https://www.melonomics.net/), Payzawat, Harukei-3, HS, and *Cucumis sativus*, *Citrullus lanatus, Benincasa hispida* (http://cucurbitgenomics.org/), and *Momordica charantia*. In the seven melon lines, three genetic markers, i.e., V432, V681, and M7, and three specific markers, i.e., Z5182, Z5184, and Z5257, were positioned. The colored boxes correspond to the main collinear blocks shown in Fig. S3

We detected *Vat*-related sequences in the seven M5-M4 regions (Fig. 1). By manual annotation, we identified two to five *Vat* homologs coding for proteins (Table S1). These homologs contained one to seven R65aa motifs in the LRR2 part and were located on both DNA strands. For PI 161375, we sequenced full coding sequences (CDSs) of its three *Vat* homologs, while for Doublon, Anso77, Piel de Sapo (for DHL92), and Payzawat we confirmed their expression by RT-PCR and validated the predicted CDSs by sequencing the junctions between the different exons (Methods S2 and Table S2). For HS and Harukei-3 we did not have RNA/DNAs to validate the predicted CDSs.

On the forward strand, we called the first *Vat* homologs *Vat-1*, located 58.8–70.9 kb from M5 according to the melon lines. These *Vat-1* contained three, four, five, or seven R65aa motifs in the LRR2 part. We called the second *Vat* homologs *Vat2*, located 77.5–137.4 kb from M5 and they contained one, two, three, or seven R65aa motifs in the LRR2 part. We kept this *Vatx* nomenclature for all *Vat* homologs identified on that strand. They were located within the first 188 kb of the M5-M4 regions. We also identified one to three *Pseudo-Vat* containing a premature stop codon or an insertion, or being only partial sequences of typical *Vat* homologs. We identified transposable elements (TEs) in 6 insertions of the 12 *Pseudo-Vat*. All TEs were retrotransposons belonging to Line-1 group, they had a similarity over 99.8% for 2540 bp. All *Vatx* and *Pseudox-Vat* located between the V432 and Z5184 markers—a region which inflated with the number of *Vatx* and *Pseudox-Vat* sequences (Fig. 1). Remarkably, the last *Vat-*related sequence located just before the Z5184 marker (113–196 kb from M5), contained a single R65aa motif in the LRR2 part in all lines but HS in which we did not identify any exon 2.

On the complementary strand, we identified one *Vat* homolog in the M5-M4 region of PI 161375, Payzawat and HS located about 60 kb before M4. We called that homolog *VatRev*, which contained a single R65aa motif in the LRR2 part. In the four other melon accessions, this was a noncoding locus due to an insertion (about 5 to 6 kb in which we identified variable TEs (integrase or transposase) and to the presence of premature stop codons—we therefore called them *Pseudo-VatRev*. The positions, annotations, and diversity of all of these *Vat*-related sequences are summarized in Table S1.

By automatic annotation (Methods S3) we identified two or five oxidosqualene cyclase genes located between the last *Vatx/Pseudox_Vat* and *VatRev/Pseudo_VatRev* (Fig. 1). Upstream of the *Vatx* homologs, the region exhibited a highly conserved gene organization with two disease resistance proteins, a serine/threonine-protein kinase (STK), and a cingulin (CGN). Downstream of the *VatRev* locus, up to M4, the region also exhibited highly conserved gene organization, with five genes (Fig. 1). The annotations and positions for all genes in the M5-M4 region are presented in Data S3 and S4 and the transcripts are presented in Data S2.

In order to investigate the Vat cluster organization in the cucurbit family, we sought to retrieve the M5-M4 region in genomes available in public databases. We focused on seven genomes obtained from long-read sequences of other cucurbit species: *Cucumis sativus* cv 9930 and Gy14 (*n* = 7)*, Citrullus lanatus* cv 97103 (*n* = 11), *Benincasa hispida* cv B227 (*n* = 12), *Cucurbita argyrosperma subsp. argyrosperma* (*n* = 20), *Luffa cylindrica* (*n* = 23), and *Momordica charantia* cv Dali 11 (*n* = 11). We searched for the highly conserved genes identified on both extremities of the M5-M4 region in melon (STK, CGN, ERAD-associated E3 ubiquitin–protein ligase, 50S ribosomal protein L18, manganese-transporting ATPase, vesicle-associated protein 2–2) and the first exon of *Vat-1*, all from PI 161375, in the full genome of each cucurbit (Data S5). We spotted at least four of the six conserved genes in a single contig or scaffold for all the species and retrieved sequences of 125–445 kb (Data S6). These sequences were located on chromosome 2 of *C. sativus* and *C. lanatus*, on chromosome 6 of *B. hispida* and on chromosome 8 of *M. charantia*. Sequences were located on scaffold 4 for *L. cylindrica* and on scaffold S335 for *C. argyrosperma*.

By manual annotation, we identified one *Vat*-related sequence with 3–4 putative exons in *C. sativus, C. lanatus*, and *B. hispida*. We only have seeds for *C. sativus* and *C. lanatus*; we confirmed expression of *Vat*-related sequences by RT-PCR in both species (Methods S2 and Table S2). For *C. lanatus*, the junctions between the different exons of the predicted CDS were validated by sequencing, nevertheless, the predicted CDS in cv 97103 contained an early STOP codon. For *C. sativus*, the first manually predicted CDSs, very close to those from *Vat* with one R65aa in melon, were not confirmed by exon junction sequencing, either in cv Gy14 or cv 9930. Actually both CDSs contained two supplemental exons, that were included in our final manual annotation. We also identified two sequences related to the first exon of *Vat-1* of PI 161375 in *M. charantia* (Fig. 1) but we did not identify any *Vat*-related sequences in *C. argyrosperma* and *L. cylindrica*. Then we conducted automatic annotation of the regions (Methods S3). Like in melon, the *Vat*-related sequences in *C. sativus*, *C. lanatus*, and *B. hispida* were located downstream of STK and Cingulin genes and followed by oxidosqualene cyclase gene(s). In *M. charantia*, the *Vat*-related sequences were located downstream of the STK gene, but Cingulin and oxidosqualene cyclase genes were absent (Fig. 1). Contrary to melon, we did not identify any *VatRev* in the region in any other cucurbits. Remarkably, in both *C. sativus* genomes, we identified another *Vat*-related sequence at the other extremity of chromosome 2, i.e., out of the Vat cluster, it was located 2.5 Mb before the end of the chromosome (Data S5 and S6). We confirmed its expression by RT-PCR in both *C. sativus* (Table S2). Manganese-transporting ATPase PDR2 and vesicle-associated protein 2–2 genes were confirmed downstream in all cucurbits. DCD domains were detected in all cucurbits except *M. charantia*, whereas ERAD-associated E3 ubiquitin–protein ligase and 50S ribosomal protein L18 were identified only in *C. sativus* and *B. hispida*. The annotations and positions of all genes in the studied region and the *Vat*-related sequences out of the Vat cluster in *C. sativus* are presented in Data S7 and S8 and the transcripts are presented in Data S6.

**Fig. 2:**
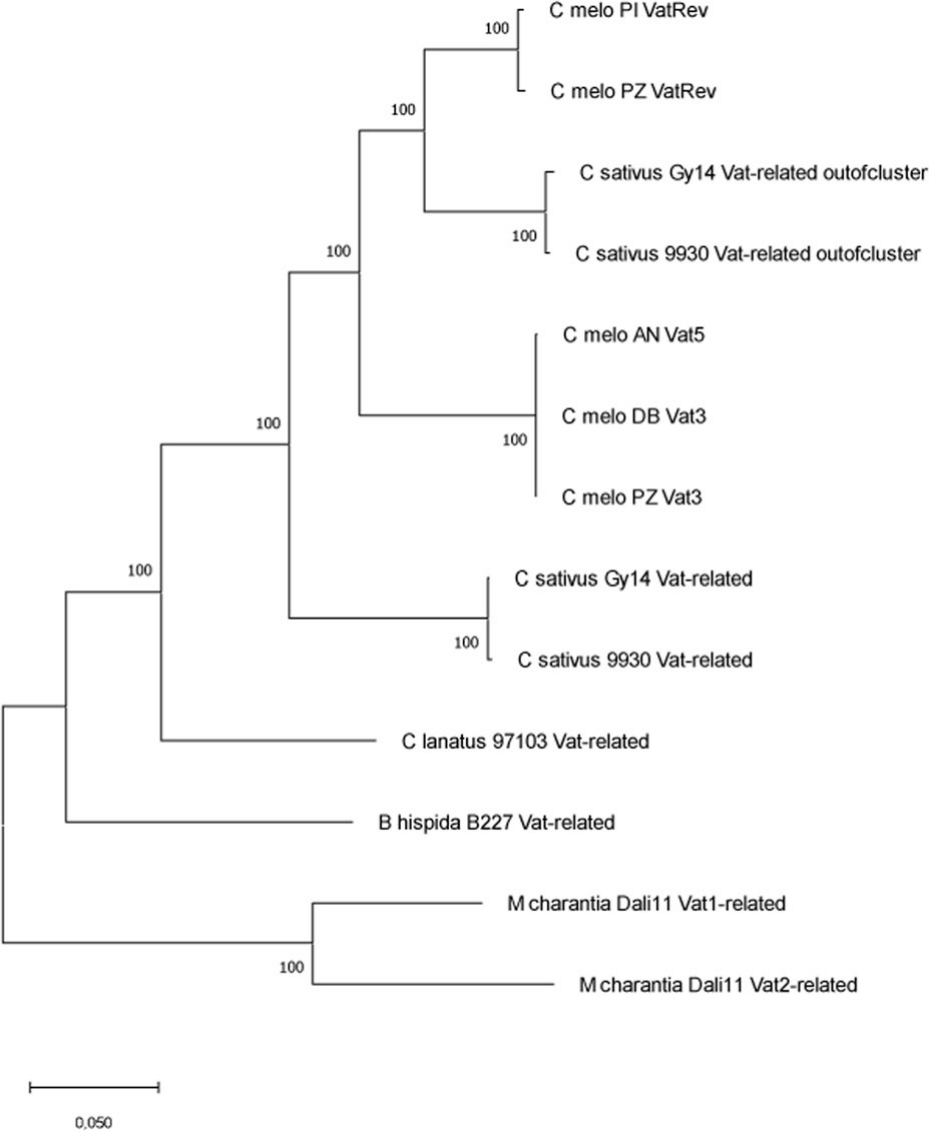
Phylogenetic tree from CDSs of *Vat*-related sequences (with one R65aa for *C. melo*) retrieved for cucurbit species. All sequences except ‘out of cluster’ sequences were retrieved from CDSs of the *Vat*-related sequences presented in Fig. 1. The MegaX software suite^43^ was then used to infer the evolutionary histories. The percentage of trees in which the associated CDSs clustered is shown next to the branches (500 replications). The tree is drawn to scale, with branch lengths measured according to the number of substitutions per site

We aligned CDSs expected from all of these *Vat*-related sequences with CDSs of the ‘simplest’ *Vat* homologs in melon, i.e., containing only one R65aa motif and we reconstructed their phylogeny (Fig. 2). All CDSs from *C. sativus* or *melo* shared a common ancestry, and the tree highlighted that the isolated *Vat* homologs in *C. sativus*, so-called ‘out of cluster’, i.e., not located between the cingulin and oxidosqualene genes, had a common ancestry with *VatRev* in melon.

### Melon diversity in the number of *Vat* homologs and R65aa in these genes

**Fig. 3:**
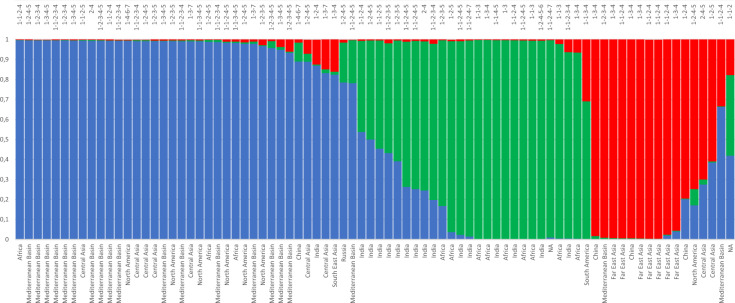
*Vat* homolog diversity in 80 melon accessions. Percentage assignation of each accession to three genetic clusters. The baseline indicated the geographical origin of the accession. The upper line gives information obtained from amplicons of the LRR2 domain: the number of digits gives an estimate of the number of homologs in the accession, each digit gives the number of R65aa estimated in each homolog. Twenty SSRs distributed over the 12 chromosomes were amplified and their diversity analyzed by Bayesian clustering^26^. The allele size at the eight microsatellite loci and probabilities of assignation at the three genetic clusters are given in the Table S3

In melon, the number of *Vat* homologs and the number of R65aa they contain is a major form of genetic diversity. We investigated both polymorphism types among 86 melon accessions from large geographical origins and botanical groups. We first characterized 80 out of these 86 accessions for their genetic diversity with 20 SSR markers (Table S3). The accessions were assigned to three clusters using Bayesian clustering. The first one, i.e., the blue cluster in Fig. 3, pooled most accessions originating from the Mediterranean Basin and Central Asia. The second one (green), contained accessions from Africa and India, most of them being wild types, and the third one (red) included accessions originating from the Far East.

We designed four pairs of primers, Z649FR, Z5895FR, Z6097F/Z6095R, and Z5474FR (Table S4), allowing us to infer the number of *Vatx* as well as any *VatRev*. We inferred 323 *Vatx* or *VatRev* in the 80 accessions (Table S3). We revealed at least four homologs in 57 lines out of the 80, thus revealing a high *Vat* duplication rate in these lines which were frequently belonging to the blue cluster (Fig. 3). We revealed a homolog with one R65aa motif, either *Vatx* and/or *VatRev*, in 78 out of the 80 accessions. Actually, *Vatx* with two or four R65aa were observed in more than two-third of the accessions while *Vatx* with three or five R65aa were observed in about half of the accessions. *Vatx* with six and seven R65aa were rare (<10%).

### Unraveling SNPs in *Vat* CDSs of 21 melon accessions

**Fig. 4:**
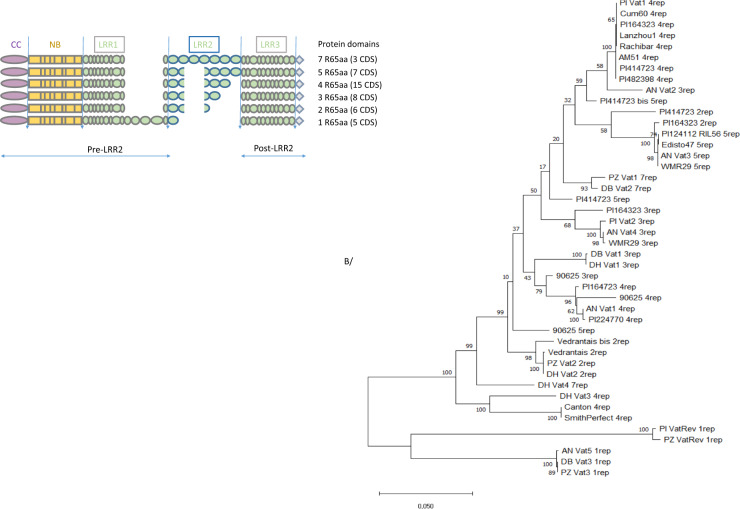
Phylogenetic tree for 44 *Vat* homologs obtained from 21 melon accessions. Each CDS was named Accession name_*Vat* number in the accession (when known) number of R65aa within the CDS. AN for Anso77, DB for Doublon, PZ for Payzawat, and PI for PI 161375. **A** Schematic representation of the multiple alignments of the 44 CDS. The alignment was independently managed for three blocks—pre-LRR2/LLR2/post LRR2—with the MUSCLE algorithm in the Seaview software package^42^. **B** The MegaX software suite^43^ was then used to infer the evolutionary histories. The percentage of trees in which the associated CDSs clustered is shown next to the branches (500 replications). The tree is drawn to scale, with branch lengths measured according to the number of substitutions per site

We gathered 44 CDSs to investigate the sequence diversity of the *Vat* homologs. First CDSs from the 19 *Vatx* and *VatRev* genes identified in the clusters described above for the five melon lines. We obtained 25 additional CDSs from 16 other melon lines (Methods S2 and Data S9). Overall, the three genetic groups of melon identified by Bayesian clustering were represented among the 21 accessions (Table S3). The 44 CDSs had 1 to 7 R65aa motifs in LRR2. Three parts, pre-LRR2/LRR2/post LRR2, were independently aligned (Fig. 4), and then the phylogeny was reconstructed. The phylogenetic tree highlighted strong differentiation between the *Vat* homologs with a single R65aa motif, which have a common ancestral gene, and all others. Homologs with a single R65aa motif had a 522–525 bp insertion at the end of the LRR1 part compared to other *Vat* homologs (Fig. 4). Explosive diversification produced homologs with two to seven R65aa motifs and, according to the five clusters described above, they probably matched the *Vatx* genes (on the forward strand).

**Fig. 5:**
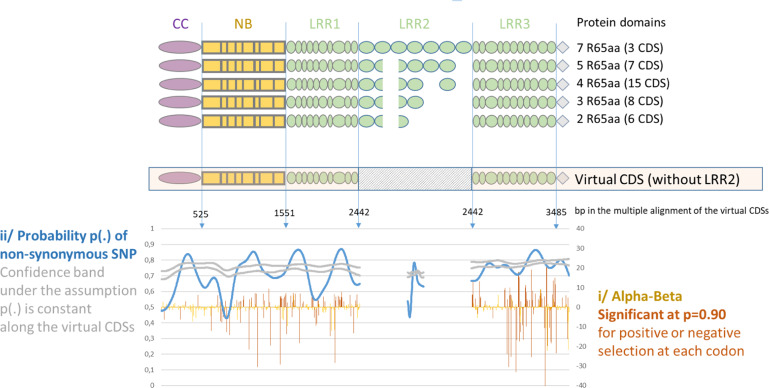
SNP analysis in 39 CDSs of *Vat* homologs with more than one R65aa. Typical alignment obtained for the CDS with different numbers of R65aa (alignment pre-LRR2, LRR2, and post-LRR2 independent), the virtual CDSs did not contain any R65aa. i The virtual CDSs were analyzed by FUBAR^44^, a Bayesian approach to infer nonsynonymous (dN) and synonymous (dS) substitution rates on a per-site basis represented by [alpha-beta]. This method assumes that the selection pressure for each site is constant throughout the entire phylogeny. ii The two parts of virtual CDS and 152 R65aa contained in the 39 CDSs were analyzed independently for the distribution of nonsynonymous SNPs, represented by the probability of nonsynonymous SNPs

We focused on the phylogenetic clade exhibiting explosive diversification and found that it contained 39 *Vat* homologs with at least two R65aa motifs. According to the phylogeny, the duplicated homologs gained and lost R65aa motifs during the diversification process. We used a Genetic Algorithm for Recombination Detection (GARD) to investigate putative gene conversion or reciprocal unequal crossover between adjacent *Vatx*. GARD searches for evidence of segment-specific phylogenies revealing recombinant sequences i.e., not adequately described with a single phylogenetic history. The R65aa motifs were excellent candidates for mispairing for both gene conversion or reciprocal unequal crossover. We built virtual CDSs without any R65aa parts to facilitate breakpoint detection in LRR2 (Fig. 5). If unequal crossover was the main process involved in inflating the R65aa motif, then we would expect to obtain evidence of a breakpoint at the junction between LRR1 and LRR3 parts. Among the 39 CDS, we used GARD to analyze the alignment of 31 virtual polymorphic CDS with the 2442 bp pre-LRR2 part and 1212 bp post-LRR2 part. The algorithm revealed up to 10 recombination breakpoints among 548 potential breakpoints, with a median distance of 359 bp. The different topologies model can be preferred over the single topology model by an evidence ratio of 100 or greater, which suggested that at least one of the breakpoints reflected true topological incongruence. Strikingly, when two breakpoints were considered, one was predicted at the LRR1-LRR2 junction (2247 bp) as would be expected if unequal crossovers occurred between R65aa.

Second, we inferred nonsynonymous (dN) and synonymous (dS) substitution rates on a per-site basis using the FUBAR Bayesian algorithm based on the assumption that selection pressure on each site was constant throughout the entire phylogeny. Significant diversifying and conservative selection was detected for codons all along the alignment of the virtual CDS (Fig. 5). Moreover, we tested if, as expected for genes belonging to the NLR family, LR domain was the most diversified domain along the Vatx proteins. For each codon, we estimated the conditional probability of observing an amino acid change when the codon changes in the set of virtual CDSs (independently considering the pre-LRR2 and post-LRR2 parts) and in the set of the 152 R65aa motifs contained in those 39 virtual CDSs (see “Experimental procedure” for details). This probability—estimated by kernel smoothing with a 20–25 bp bandwidth for the pre-LRR2 and post-LRR2 parts and 8 bp for the LRR2 part—exhibited significant peaks and valleys (Fig. 5). Nonsynonymous codons were more frequent than expected in two parts of the LRR1 region and one part of the LRR3 region, and remarkably in the R65aa motif, so the frequency of these nonsynonymous codons putatively increased with the number of R65aa motifs in the LRR2 part.

## Discussion

**Fig. 6:**
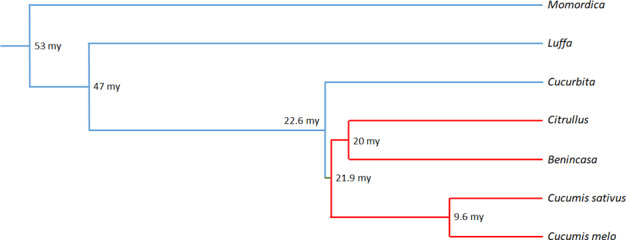
Topology of Cucurbitaceae phylogeny retrieved from the Cucurbitaceae tree http://timetree.org/. The clade in red corresponds to the Benincaseae tribe

Cucurbitaceae have been evolving over the last 60 million years (Fig. 6) from an ancestral Cucurbitaceae with 12 protochromosomes^11^. The Benincaseae species (in red in Fig. 6), including melon, cucumber, and watermelon, diverged from Cucurbiteae species 22.6 mya (see http://timetree.org/). Then allotetraploidization, or whole-genome duplication, occurred in the different *Cucurbita* ancestral species^12,13^. We investigated in several cucurbits the R gene cluster spanning the *Vat* gene belonging to the NLR gene family. Note that NLR genes have often been mispredicted by automatic annotation tools due to their duplicated and clustered nature, leading to fragmented or absent annotations, or otherwise they are sometimes annotated as repetitive sequences^14^. Automatic annotation of *Vat*-related sequences in *C. lanatus* exemplified this problem: in both assemblies in public databases, cv Charleston gray and 97103, the genomic sequences were 100% identical but automatic annotation identified either one or two genes, but none complete Vat-related sequence. For *Vat* genes in melon, these difficulties have been exacerbated by the presence of a 65aa pattern repeated a variable number of times in the different homologs present in the cluster. We therefore used genomes derived from long-read sequences to retrieve *Vat* homologs borne by the different cucurbit species or lines in melon, and we developed a tailored BLAST/annotation strategy.

*Vat*-related CDSs were identified in the *Citrullus*, *Benincasa*, and *Cucumis* genera belonging to the Benincaseae tribe while we did not spot any *Vat*-related CDSs in *Cucurbita* or *Luffa* species. The microsynteny between genomic regions spanning *Vat* homologs in *C. melo*, *C. sativus*, *C. lanatus*, and *B. hispida* suggested a common ancestor of <22 my old for this region in the Benincaseae tribe. The simplest region we could imagine in the tribe successively displayed genes coding for a serine/threonine-protein kinase, Cingulin, *Vat*-related-one R65aa, oxidosqualene cyclase, Development Cell Death domain protein, manganese-transporting ATPase, and vesicle-associated protein. It was located on chromosome 5 in melon and on its syntenic chromosome 2 in cucumber and watermelon and on chromosome 6 in wax gourd. Some of these genes were also identified within a short genomic region in *Luffa* and *Cucurbita*. A model of the paleohistory of R gene clusters suggested that ancestral R loci duplicated with the whole-genome duplication common to eudicots, and then deleted or selected R genes in sensitive or dominant chromosome pairs^1^. In the dominant chromosome, R genes should subsequently be subject to transposition and clusterization. According to that scheme, a *Vat*-related gene should be present in a cucurbit protochromosome. In an older cucurbit, i.e., *M. charantia* that diverged from other lineages more than 50 mya (Fig. 6), we spotted two sequences related to the first exon of *Vat* between an STK and a manganese-transporting ATPase followed by a vesicle-associated protein. The presence of these two sequences in *M. charantia* suggested that a *Vat*-related gene was present in the ancestral cucurbit older than 60 my, lost in *Luffa* and *Cucurbita* lineages but conserved in the Benincasae lineage.

In the *Cucumis* genus (about 18 my old), the region has expanded by tandem duplication of oxidosqualene cyclases, particularly in *C. sativus* that contains up to seven oxidosqualene cyclases. These enzymes led to simple triterpenes which are components of surface waxes and specialized membranes and might potentially act as signaling molecules^15^. Following the inflation of oxidosqualene cyclases, the *Vat* homologs sharply expanded in *C. melo*, which diverged from the lineage behind *C. sativus* 9.6 mya. In *C. melo*, we identified two *Vat*-related sequences with a single R65aa, one on the forward strand (*Vat* or *Pseudo-Vat*) and one on the reverse strand (*VatRev* or *Pseudo-VatRev*). *VatRev* was notably absent in *C. sativus*, *C. lanatus*, and *B. hispida*. Surprisingly, *C. sativus* contained a *Vat* homolog with one R65aa at the other extremity of the chromosome carrying the Vat cluster, whereas this homolog was absent in *C. melo*, *C. lanatus*, or *B. hispida*. This ‘out of cluster’ *Vat* homolog in cucumber and *VatRev* in melon shared a common ancestry (Fig. 2). They might have evolved from a retrotransposition just after the two lineages diverged: ‘copy’ of an ancestral *Vat* with a single R65aa and ‘paste’ at the other extremity of chromosome 2 in cucumber and on the reverse strand in the Vat cluster in melon.

Thereafter we highlighted the all-out duplication of *Vat* homologs, with some new genes becoming pseudo genes later, while building the Vat cluster in melon. Transposable elements probably played an important role in shaping the number of *Vat* homologs in the cluster, as it is now clear that all transposon superfamilies are capable of duplicating genes or gene fragments^16^. In melon, a recent burst of transposable elements occurred^17^ and, in accordance, TEs in *Pseudo-Vat* homologs shared high similarity suggesting they are recent and active (Table S1). The inflation of *Vat* homologs occurred upstream of the pair of head-to-head oriented *Vat* homologs (Fig. 1). We highlighted duplication in all wild melons which were studied, thus suggesting that the duplication process started before domestication. This phenomenon was particularly prevalent in the genetic group of melons belonging to the blue cluster in Fig. 3 (4.3 homologs per accession on average). *C. melo* originated from Southeast Asia^18^, so *Vat* homolog inflation in the Mediterranean Basin could be related to an adaptation to this new environment in response to the pathogens present. Remarkably, *Vat* homolog inflation occurred concomitantly to *Aphis gossypii* differentiation from its sister species (*Aphis frangulae*) estimated at 5 Mya (timetree.org^19^). *A. gossypii*, currently called the cotton-melon aphid, is a pest for several crops, and it now seems quite clear—based on genetic, ecological, and lab experiment data—that some *A. gossypii* aphids have specialized on cucurbits^20^.

Diversification of the newly generated *Vat* gene copies in melon occurred via two processes. First, SNPs emerged all along the gene, and we highlighted a pattern of successive ‘hills and valleys’ for nonsynonymous SNPs. Second, we revealed inflation of R65aa motifs in the LRR2 part, given that we observed up to seven R65aa repeats in a single gene. LRR copy number variation was described quite long ago at the Cf-x locus in tomato, thus conferring resistance to *Cladosporium fulvum*^21^. In melon, the phylogeny of the *Vat* homologs suggested that the R65aa gain/loss occurred during the diversification process (Fig. 4) and ultimately homologs with two to five R65aa were the most frequent (Table S3). Actually, reciprocal unequal crossovers or unidirectional gene-conversion events may have generated this diversity by virtue of the high degree of homology between R65aa motifs. In flax, unequal crossover at the M locus—an NLR gene—occurred in a 45 bp region that is invariant between LRR repeats of five lines^22^. The successive R65aa motifs in *Vat* homologs revealed a candidate for this phenomenon, and we detected a putative breakpoint in this genomic area. Besides the R65aa number variation, we showed that SNPs were more frequently nonsynonymous compared to other SNPs between codons 25 and 35 in these R65aa motifs.

To date, no phenotype has been associated with these *Vat*-related genes in any cucurbits except two *Vat* genes in *C. melo*. One corresponds to *Vat-1* with four R65aa in PI 161375, which confers resistance to virus when inoculated by *A. gossypii*, in addition to resistance to *A. gossypii*^4^. The second one, i.e., *PmW*, corresponded to the CDS WMR29_5rep in our set and confers resistance to powdery mildew (*Podosphaera xanthii*)^8^. Then, the Vat cluster was found to share different features with the Mi cluster in tomato, i.e., both conferred resistance to aphids and powdery mildew, but resistance to nematodes and whiteflies was only observed for the Mi cluster^23^. Like melon lines for the Vat cluster, tomato lines were reported to contain different *Mi*-homolog compositions, with homologous blocks, but with complex differences in arrangement thought to have contributed to the evolution of recognition^24^. Then the number of homologs is a decisive polymorphism for pathogen recognition. Accordingly to both phenotypes known in melon, the number of R65aa motifs could also be a decisive polymorphism for pathogen specificity recognition, as shown in flax, where the loss of a repeat unit of the LRR domain within the M locus was found to be associated with inactivation of resistance to flax rust^22^. Nevertheless in melon, SNPs are probably also involved in pest/pathogen specificity recognition. Actually, the combination of SNPs, the number of R65aa motifs in *Vat* homologs and the number of *Vat* homologs is potential evidence of a very broad recognition range in the Vat cluster.

Because several other resistance loci have been genetically located near *Vat*—resistance to the cucumber vein yellowing virus, to *Fusarium oxysporum* f. sp. *Melonis* or the *Fn* gene which triggers plant necrosis in response to some ZYMV isolates^6^—and several putative disease resistance genes included in the M5-M4 genomic region, this region is of great interest for melon breeding programs, with the aim of associating alleles conferring resistance to pests and pathogens. Research for new resistances should focus on Mediterranean accessions, which offer the most diversified Vat clusters. Surprisingly, the region was not located on the same arm of chromosome 5 in Payzawat and the six other lines, suggesting a translocation which may reduce the recombination potential. However, no long reads supported the assembly of Payzawat at the breakpoint, and translocation of the chromosome 5 extremity is still putative.

## Experimental procedure

### Characteristics of the melon accessions

Eighty-six melon lines were used and are listed in Table S3 with their geographic origins and botanical groups. Genomic DNA was isolated from fresh young leaves using the DNeasy Plant Mini Kit (QIAGEN, www.qiagen.com). Twenty microsatellite loci distributed in the 12 linkage groups of melon^25^ were amplified at the Gentyane platform and the allele sizes were inferred by GeneMapper v3.7 software (APPLIED BIOSYSTEMS, Foster City, California, USA). The lines were assigned to genetic clusters by Bayesian clustering^26^. For 1–10 clusters, we compared 10 replicate runs (admixture model with a burn-in of 250,000 iterations and a subsequent Markov chain of 500,000 iterations) and, according to the Evanno method^27^, the most likely number of genetic clusters was three. We performed one run of the admixture model with a burn-in of 500,000 iterations and a Markov chain of 1,000,000 iterations to infer the probability of assignment at three clusters of each line.

### Producing/predicting CDS from *Vat* homologs (*C. melo*) and *Vat*-related sequences (other cucurbits)

Producing processes of CDS are detailed in Methods S2.

For PI 161375, *Vat-1* and *Vat2* full cDNAs were amplified by long-range PCR with Z717F and R primer located in 5′ and 3′-UTR (Table S2). For the melons Doublon, Anso77, Piel de Sapo T111 (for DHL92), and Payzawat, the cucumbers cv 9930 and Gy14-specific primers of each *Vatx* homolog (Table S2) were designed in the predicted exons. Partial cDNAs spanning all exon junctions, were amplified by RT_PCR and directly sequenced (without cloning) by SANGER technology. For 16 other *C. melo* lines, we predicted CDSs from their genomic sequences by homology with CDS of *Vat-1* from PI 161375.

### Producing amplicons from *Vat* homologs in melon diversity

We designed four pairs of primers, Z649FR, Z5895FR, Z6097F/Z6095R, and Z5474FR (Table S4), partly amplifying *Vatx* and *VatRev*. The Z649FR primers amplified LRR2 of any *Vatx* containing more than a single R65aa motif in the seven lines except for *Vat-4* in DHL92. Z649FR primers were located in introns on each side of exon 2 spanning R65aa motifs in the LRR2 part. Amplicons obtained for the 86 melon lines underwent gel electrophoresis. Band numbers observed for each line corresponded to the minimal number of *Vat* homologs with more than one R65aa motif; band sizes allowed us to infer the number of R65aa motifs in these *Vat* homologs.

For *Vatx* with one R65aa and *VatReV*, because of their divergence from other *Vatx*, we designed specific primers. The Z5895FR primers, located in exon 1 and exon 2 produced an amplicon of 1502 bp on *Vatx* with one R65aa and an amplicon of 3847 bp when an insertion occurred (*Pseudo-Vatx*). To check if these homologs contained only one R65aa, we designed Z6097F at the beginning of the exon 2 and Z6095R at the end, producing an amplicon of 467 bp. The Z5474FR was designed to partly amplify any *VatRev*, it located at the end of exon 1 and produced amplicons with 933 bp. These three pairs of markers produced expected amplicons for the seven lines.

From all information we inferred the number of *Vatx* and *VatRev* in 80 melon lines and the number of R65aa motifs they contained. The number of *Vatx* and *VatRev* was underestimated if some *Vat* homologs had the same number of R65aa in a line or if any SNP occurred at primer loci of any *Vatx* or *VatRev*.

### Retrieving and annotating the genomic region spanning the Vat cluster in cucurbits

We retrieved nucleic sequences between the M5-M4 markers from the Anso77 and Doublon genome assemblies we produced, and from public databases for DHL92, Payzawat, Harukei-3, and HS genomes (FTP1, FTP2, FTP3, FTP4)^17,28–30^. To that end, we used informations from PI 161375: eight markers (M5, Z5182, V432, V681, Z5184, M7, Z5257, M4), exon 1 sequence from *Vat-1* and a set of six gene sequences (STK, CGN, ERAD-associated E3 ubiquitin–protein ligase, 50S ribosomal protein L18, manganese-transporting ATPase, vesicle-associated protein 2–2). Using blastn (NOBLAST version 2.1, blastall with E-value < 1E10^–5^) we examined sequences with a percentage score, i.e., either higher than 30 queries or a target alignment, and an identity of > 40^31^. Moreover, nucleic sequence sets were mapped against the genome assemblies using the Minimap2 alignment program for genomic DNA^32^.

According to the analyses conducted for *C. melo*, we used the same set of sequences to search similar regions in public genomes of *Cucumis sativus* cv 9930 and Gy14 (FTP5, FTP6)^33^*, Citrullus lanatus* cv 97103 (FTP7)^34^, *Benincasa hispida* cv B227 (FTP8)^35^, *Cucurbita argyrosperma* (FTP9)^36^, *Luffa cylindrica* (FTP10)^37^, and *Momordica charantia* (FTP11)^38^. Mapping and BLAST parameters were the same as in the previous step. Genome assemblies from public databases, annotation sets, and fasta files for protein and cDNA were downloaded from the Genbank NIH genetic sequence database, Melonet DB (https://melonet-db.dna.affrc.go.jp) and from the International Cucurbits Genomics Initiative (ICUGI) server CuGenDB (http://cucurbitgenomics.org/). We retrieved genomic sequences spanning all or at least some of these genes.

The genomic sequences were exported to CLC Main workbench software (V 8.1.2, QIAGEN Aarhus A/S, www.qiagenbioinformatics.com) for manual annotations of Vat-related sequences. The *Vat-1* gene from PI 161375 was used as a reference sequence to find the set of *Vat*-related sequences using the BLASTn-NCBI Basic Local Alignment Search Tool (https://blast.ncbi.nlm.nih.gov). BLAST queries with the first and third exon, corresponding respectively to the CC-NBS-ARC-LRR1 and LRR3 domains, revealed *Vat*-related sequences which were manually annotated for exons, introns, and LRR2 characteristics in reference to *Vat-1* from PI 161375. In addition, an automatic annotation was carried on (Methods S3).

### Microsynteny, phylogeny, and diversity analyses

Syntenic blocks were identified in chromosome 5 (carrying the *Vat* gene) of DHL92 and Payzawat using the Mauve multiple genome alignment software package (version 2.4.0)^39^ with default parameters. We revealed a putative transposition in that chromosome between DHL92 and Payzawat. Then we checked the contiguity of the assembly of each line at the putative transposition point by mapping the available reads for each line on its chromosome 5 sequence^40^.

For the M5-M4 genomic sequences spanning *Vat*-related sequences in melon, we produced dot plots using the LASTZ version 1.04.00 alignment tool^41^. The parameters used were as follows: --notransition --step=20 --gfextend --gapped --chain --matchcount=3000 --identity=80. Syntenic blocks in M5-M4 sequences were investigated using MAUVE with the following parameters: --weight=90000 and --seed-weight=13.

CDSs retrieved from *Vat*-related sequences in several cucurbit genomes were aligned with the MUSCLE algorithm in Seaview software^42^. For melon, only CDSs from *Vat* homologs with one R65aa were introduced in the alignment. The MegaX software suite^43^ was then used to infer the evolutionary histories. Initial tree(s) for the heuristic search were obtained automatically by applying Neighbor-Join and BioNJ algorithms to a matrix of pairwise distances estimated using the Tamura–Nei model for CDSs of *Vat*-related sequences (with one R65aa for *C. melo*) retrieved for cucurbit species and using the JTT model for *Vat* homologs obtained from 21 melon accessions. The topology with highest log-likelihood value was selected.

CDSs retrieved from *Vat*-related sequences in melon were aligned while considering three independent blocks—pre-LRR2/LLR2/post LRR2—with the MUSCLE algorithm in the Seaview software package^42^. Phylogeny was then inferred as for CDS from cucurbits. Nonsynonymous (dN) and synonymous (dS) substitution rates on a per-site basis were inferred by FUBAR^44^ based on the assumption that pervasive selection pressure for each site is constant along the entire phylogeny.

Moreover, we investigated the relative frequency of nonsynonymous SNPs along *Vat*-related sequences as described in Methods S4.

## Supplementary information

Figure S1 PI 161375 BACs clone assembly. PI-Contig 1: seven BACs clones (89 to 153 Kb); 8-80 kb overlap with 99 to 100% identity. PI-Contig 2: two BACs clones (98 and 137 Kb); 35 Kb overlap with 99% identity. PI-Contig 3: two BACs clones (72 and 113 Kb); 20 Kb overlap with 99% identity.

Figure S2 Mauve alignment [36] of chromosome 5 of DHL92 and Payzawat. Local collinear blocks are represented by blocks of the same color. The M5-M4 regions - spanning *Vat* - are located in the pink bubble.

Figure S3 Microsynteny analysis of M5-M4 sequences spanning *Vat* obtained from seven melon lines. A/ Self dot plots [41]. B/ Mauve alignment [39] of the M5-M4 regions in reference to the DHL92 M5-M4 sequence. Local collinear blocks are represented by blocks of the same color connected by lines.

Figure S4 Gel picture obtained from electrophoresis of *Vat*-homolog amplicons. A/ The amplicons were obtained by PCR using Z649 F and R primers located in the introns on each side of the exon 2 that spans the R65aa motifs in the LRR2 part of *Vat*. Band numbers observed for each line corresponded to a minimal number of *Vat*-homologs in the line; band sizes allowed to infer the number of R65aa motifs in these *Vat*-homologs. B/ The amplicons were obtained by PCR using Z6097F and Z6095R primers located at the beginning and the end of exon 2; they were specific to *Vatx* with one R65aa, and produced amplicons with 467 bp.C/ The amplicons were obtained by PCR using Z5474 F and R primers located at the end of exon 1 of *VatRev*; they produced amplicons with 933 bp when *VatRev* was present.

Table S1 Manual annotation of *Vat*-homologs sequences located in the M5-M4 region for seven melon genotypes. The *Vat-1* gene from PI 161375 was used as a reference sequence to find the set of *Vat-*related sequences by BLAST. Positions of the different *Vat*-homologs and *Pseudo-Vat* are indicated relative to M5. The transposable elements present in the insertions of *PseudoX_Vat* have been annotated after BLASTX on NCBI.

Table S2 Primers used for RT-PCR and for CDS sequencing of the different *Vat*-homologs in five melon lines and two other Cucurbits. F: forward - R: reverse. In bold: primer pairs for RT-PCR. In italic: (1) *PI_Vat-1* specific primer; (2) *PI_Vat-2* specific primer

Table S3 Information collected for the 86 lines of melon used in this study. Geographic origin, botanic group, number of R65aa motifs identified in *Vatx* i/ from cluster sequencing (5 lines), ii/ from putative CDS expected from long sequencing (16 lines), iii/ from the number of amplicons spanning the R65aa motifs, and, the probability of assignment to three most likely genetic clusters obtained after bayesian analysis of 20 SSRs markers, alleles at 20 SSRs markers.

Table S4 Primers used for long-range PCR and genomic DNA sequencing of the different *Vat* homologs in melon lines. F: forward, R: reverse.

Methods S1 Genomic resource production for PI 161375, Anso77 and Doublon and de novo genome assemblies for Anso77 and Doublon. High molecular weight (HMW) genomic DNA production and NGS sequencing with PacBio (for PI 161375 and Doublon) and Oxford Nanopore Technologies (for Anso 77 and Doublon). The genome assemblies were performed using canu v.1.8 (for Anso77 and Doublon).

Methods S2 Producing/predicting CDS from *Vat* homologs (C. melo) and *Vat*-related sequences (other cucurbits). For PI 161375, full cDNAs of *Vat1*, *Vat2* and *Vat-Rev* were amplified by RT-PCR and sequenced. For other *C. melo* (Doublon, Anso77, Payzawat) and for *C. sativus* and *C. lanatus* only partial cDNAs spanning exon junctions were amplified and sequenced.

Methods S3 Automatic annotation of retrieved genomic areas. Structural annotation using MAKER v3.01.02 software and functional annotation using InterProScan and SwissProt databases.

Methods S4 Relative frequency of nonsynonymous SNPs along *Vat*-related sequences Probability of nonsynonymous mutation p(i), that is assumed to be continuous along the sequence and confidence band using an homemade permutation method.

Data S1 Result of blast and mapping sequences from PI 161375 against *Cucumis melo* genome assemblies of Doublon, Anso77, DHL92, Payzawat, Harukei-3 and HS. The sequences used from PI 161375 were eight markers (M5, Z5182, V432, V681, Z5184, M7, Z5257, M4), exon 1 sequence from *PI_Vat1* and a set of six gene sequences (STK, CGN, ERAD-associated E3 ubiquitin-protein ligase, 50S ribosomal protein L18, Manganese transporting ATPase, Vesicle-associated protein 2-2).

Data S2 Nucleotide sequences of *Cucumis melo* including seven M5-M4 regions and all transcripts contained in these M5-M4 regions. All data for PI 161375, Doublon, Anso77, DHL92, Payzawat, Harukei-3 and HS

Data S3 Gene ontology annotation (InterproScan) for all genes in the region M5-M4 of seven *Cucumis melo*. Cv PI 161375, Doublon, Anso77, DHL92, Payzawat, Harukei-3 and HS

Data S4 Gene annotation for the region M5-M4 of seven *Cucumis melo*. Cv PI 161375, Doublon, Anso77, DHL92 and Payzawat, Harukei-3 and HS. The annotation was manual for the *Vat*-related sequences and was conducted using MAKER for other genes.

Data S5 Result of blast and mapping sequences from PI 161375 against genome of several cucurbits. *Cucumis sativus* cv 9930 and Gy14, *Citrullus lanatus* cv 97103, *Benincasa hispida* cv B227, *Cucurbita argyrosperma* Subsp *argyrosperma*, *Luffa cylindrica* and *Momordica charantia* cv Dali 11. The sequences used from PI 161375 were eight markers (M5, Z5182, V432, V681, Z5184, M7, Z5257, M4), exon 1 sequence from *PI_Vat1* and a set of six gene sequences (STK, CGN, ERAD-associated E3 ubiquitin-protein ligase, 50S ribosomal protein L18, Manganese transporting ATPase, Vesicle-associated protein 2-2).

Data S6 Nucleotide sequences corresponding to the cluster region from six cucurbits, all transcripts contained in these M5-M4 regions and transcripts of *Vat*-related out of cluster from *C. sativus*. *Cucumis sativus* cv Gy14 and cv 9930, *Citrullus lanatus* cv 97103, *Benincasa hispida* cv B227, *Cucurbita argyrosperma* Subsp *argyrosperma*, *Luffa cylindrica* and *Momordica charantia* cv Dali 11.

Data S7 Gene ontology annotation (InterproScan) for all genes in the cluster region of six cucurbits.*Cucumis sativus* cv Gy14 and cv 9930, *Citrullus lanatus* cv 97103, *Benincasa hispida* cv B227, *Cucurbita argyrosperma* Subsp *argyrosperma*, *Luffa cylindrica* and *Momordica charantia* cv Dali 11.

Data S8 Gene annotation for the cluster region of six cucurbits. *Cucumis sativus* cv Gy14 and 9930, *Citrullus lanatus* cv 97103, *Benincasa hispida* cv B227, *Cucurbita argyrosperma* Subsp *argyrosperma*, *Luffa cylindrica* and *Momordica charantia* cv Dali 11. The annotation was manual for *Vat*-related sequences and was conducted using MAKER for other genes.

Data S9 Forty-four CDSs of Vat homologs from 21 melon lines. Used to build the phylogeny tree in Figure 4.

## Data Availability

Sequencing data for genome assemblies and contigs sequences were deposited in the NCBI database under the following numbers: Bioprojects PRJNA662717 and GenBank MW591952 for Anso77, BioProject PRJNA662721 and GenBank MW591951 for Doublon, and BioProject PRJNA664659 and GenBank MW591953, MW591954, MW591955 for PI 161375.
