# Supplementary material for: Building a cluster of NLR genes conferring resistance to pests and pathogens: the story of the Vat gene cluster in cucurbits
Source: Hortic Res. 2021 Apr 1;8:72. doi: 10.1038/s41438-021-00507-0 (PMC8012345; doi:10.1038/s41438-021-00507-0)
Supplement: Supplementary file 1 — Figure S1 PI 161375 BACs clone assembly. PI-Contig 1: seven BACs clones (89 to 153 Kb); 8-80 kb overlap with 99 to 100% identity. PI-Contig 2: two BACs clones (98 and 137 Kb); 35 Kb overlap with 99% identity. PI-Contig 3: two BACs clones (72 and 113 Kb); 20 Kb overlap with 99% identity. [file 41438_2021_507_MOESM1_ESM.pdf]

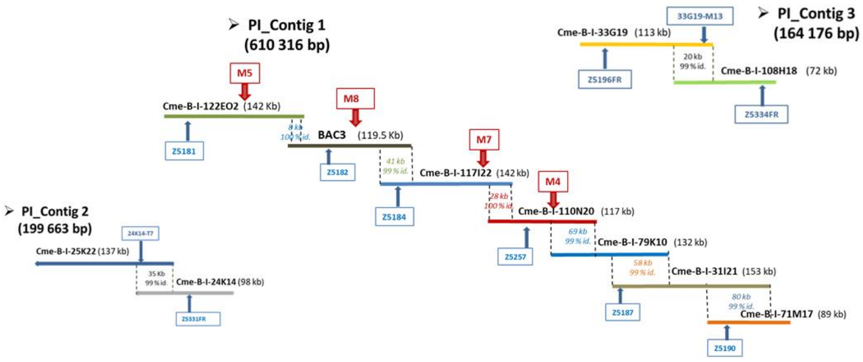

Contig	Markers	Primers sequences (5'-3')	Amplicons size (bp)
2	24K14-T7	F: TGGTGGCACGAAATTTGTTA<br>R: AGACTTGCGGCTAAGGAACA	374
	Z5331	F : GATGAACAGATGAGAGTGTGTGG<br>R : AAAGCGGAGAGATTGGATTGGA	224
1	Z5181	F : ATCTACGTCAAGTGATTATTTTCTGCT<br>R : AAAGCGGAGAGATTGGATTGGA	433
	M5	F : TTAACGCTCTTTGTTCTAAGCTTTTGTA<br>R : CAACCATTGTATGATGCAAATGACCAA	156
	Z5182	F : TCGTCAAAGGACCCCAAA<br>R : TAAACGAAAATAGGTAGCAGGTAAGAA	275
	V432	F : TTAGAGTGGCAAAGGGAAGATGGG<br>R : AACTTCTCCAACTCCCTCCACTGC	432
	V681	F : GTTGTATATGGCTTCCCTGTAGCC<br>R : GGAATCTTGTTGAGGCCGAGAGGG	681
	M8	F : TCGTGAAGGGTTTTGGAGAGTGAGAAA<br>R : CAACAACGCGTCGGGAGATGCGTCGG	228
	Z5184	F : CCCAAAAGAATCCAAAGAGATG<br>R : CTTGGGGATGAAATGATATAAAGTG	298
	M7	F : GCAAATGTTTTCCCATAAGTTC<br>R : CCTCTAGCTACATTACGTACG	347
	Z5257	F : AATGATTAAGAACCCCAGTTTGATAG<br>R : CAAGAAGTTATGTAGCTGATGCTGA	379
	M4	F : GGCTGACAGTTAGAGTAACCA<br>R : TAATAACATGAAACACTCTGTCAATTT	207
	Z5187	F : AAAAACATACTTGTTCAAACCCTACC<br>R : ACAGATCAATCCCAAGAGTAAAA	366
	Z5190	F : CCTAAAATCACCCATAAACACCA<br>R : TATTAACTACTCTTCTTCAACCCTCCA	456
3	Z5196	F : GTTTCTGGGAGTGACGTGGT<br>R : GTGGTGGTTTGACTGGAAGAA	652
	33G19-M13	F : ATCTTGATGCCGATGAGGAC<br>R : TTTTCTCCCGTAACCACGTC	247
	Z5334	F : CTGATGTCGGTTGGCTGGTT<br>R : TCTCGCGCCACTCTCATAAC	337
