# Supplementary figures and images for: Building a cluster of NLR genes conferring resistance to pests and pathogens: the story of the Vat gene cluster in cucurbits
Source: Hortic Res. 2021 Apr 1;8:72. doi: 10.1038/s41438-021-00507-0 (PMC8012345; doi:10.1038/s41438-021-00507-0)

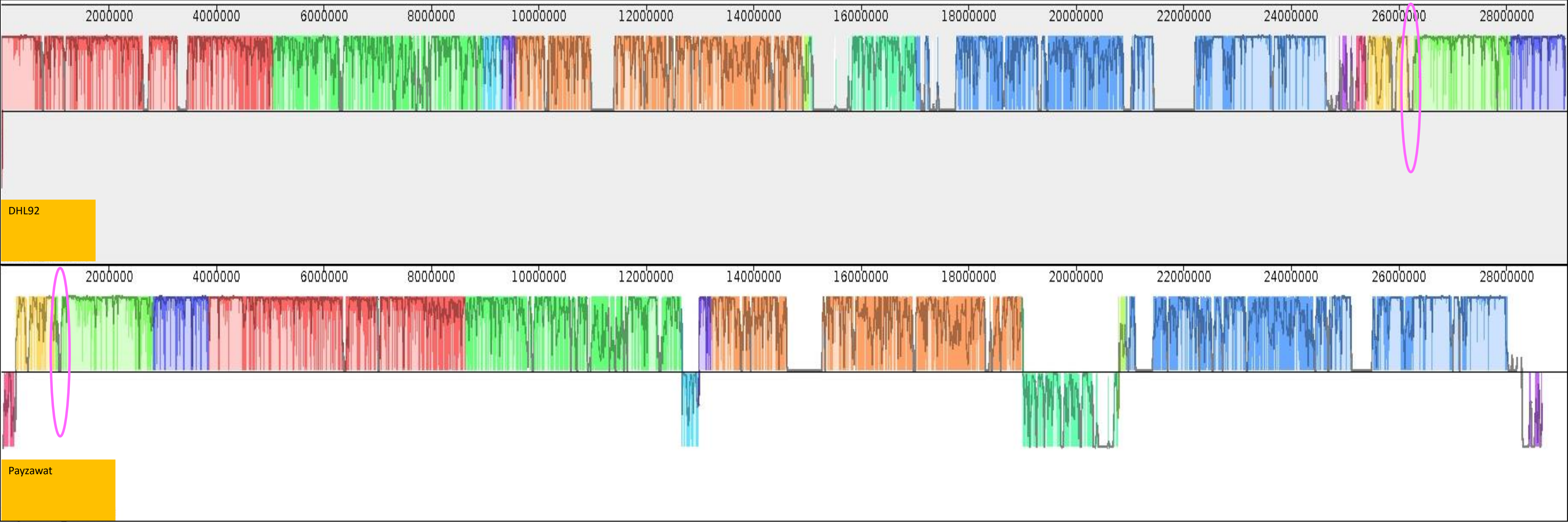

Supplement: Supplementary file 2 — Figure S2 Mauve alignment [36] of chromosome 5 of DHL92 and Payzawat. Local collinear blocks are represented by blocks of the same color. The M5-M4 regions - spanning Vat - are located in the pink bubble. [file 41438_2021_507_MOESM2_ESM.pdf]

A/

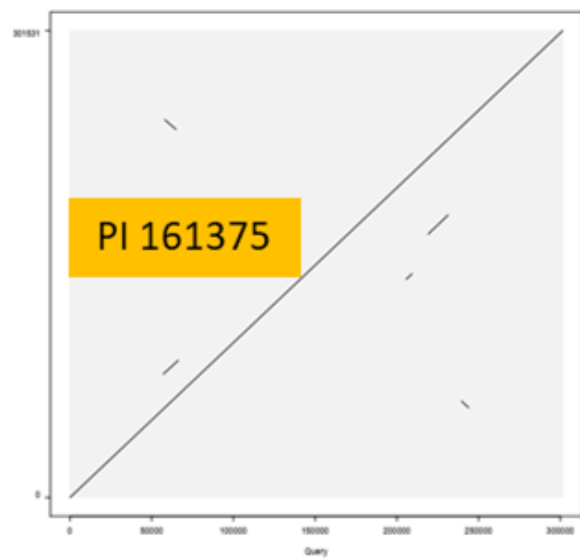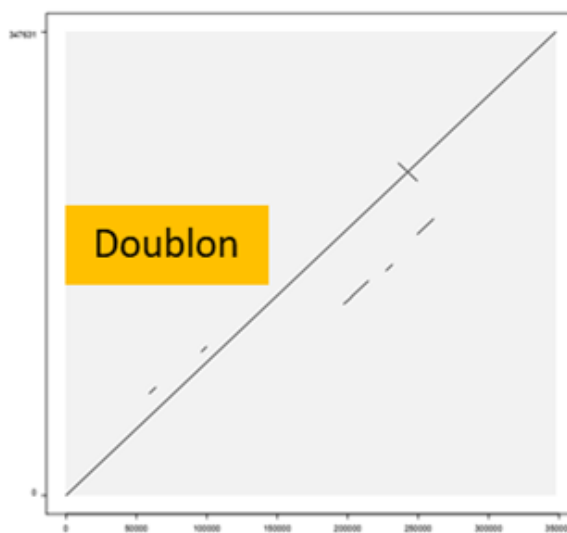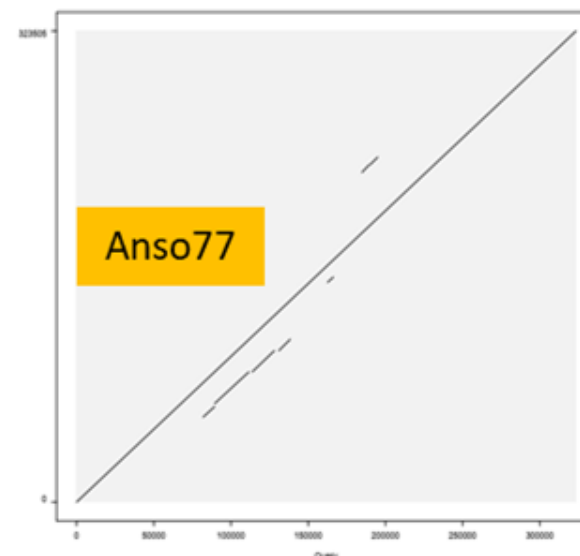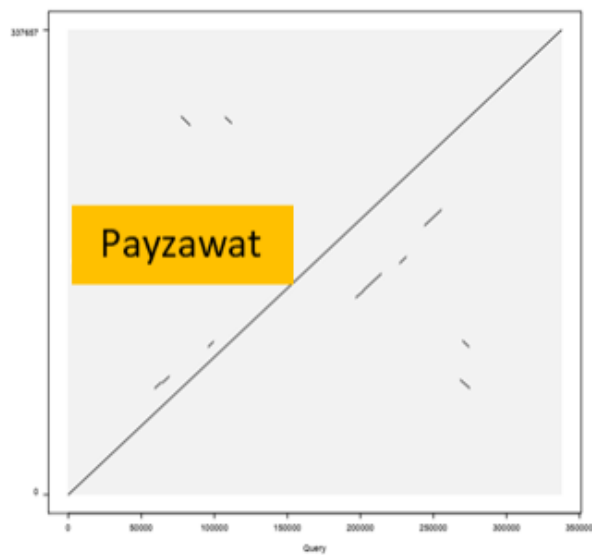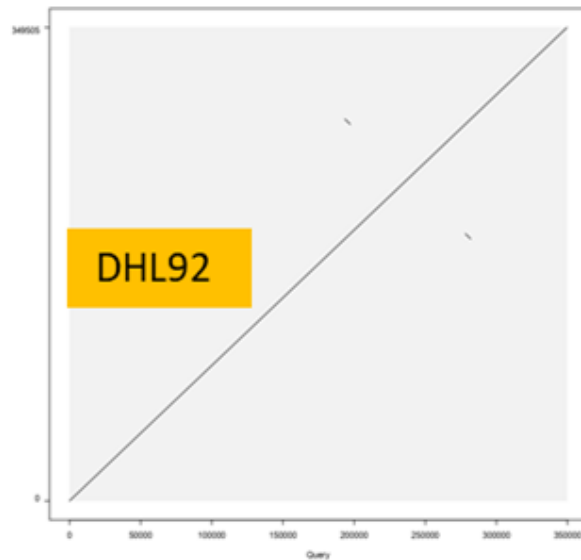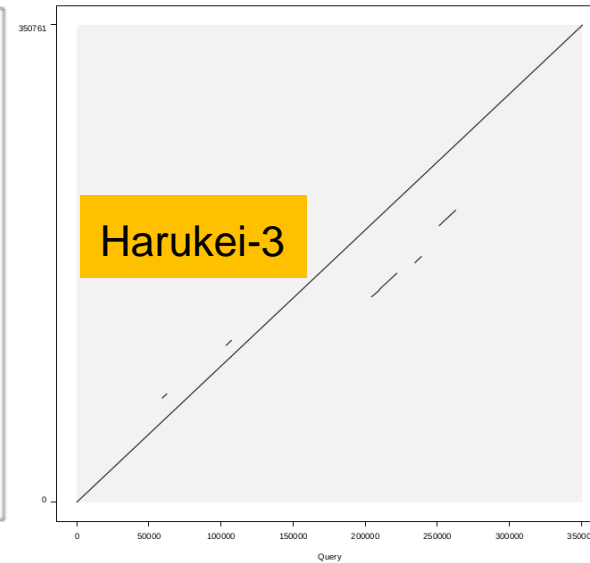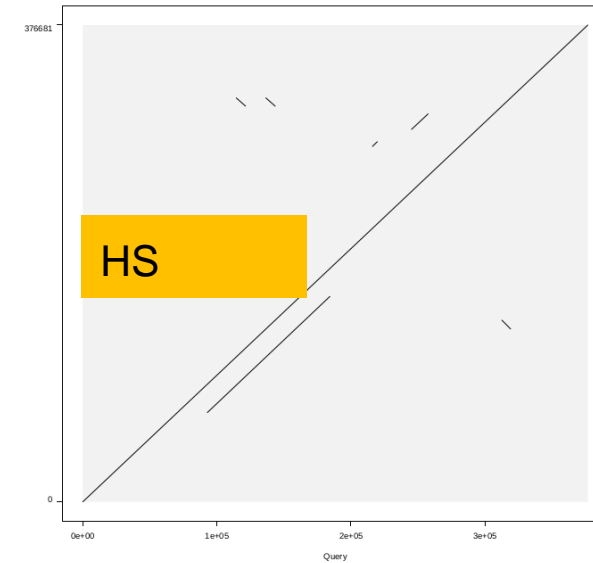

B/

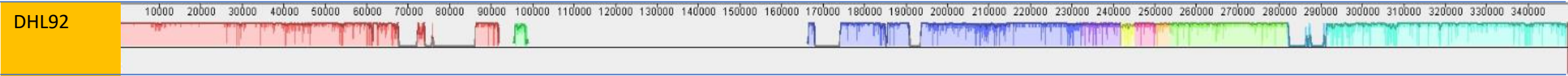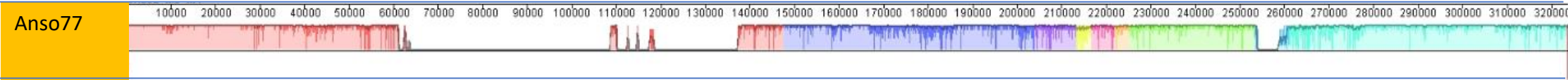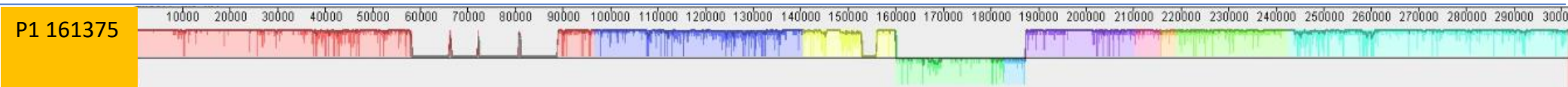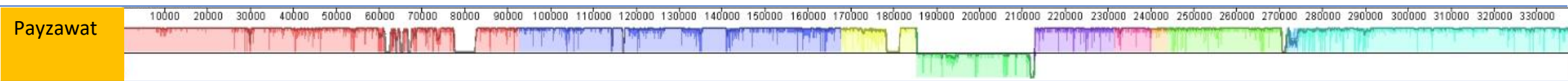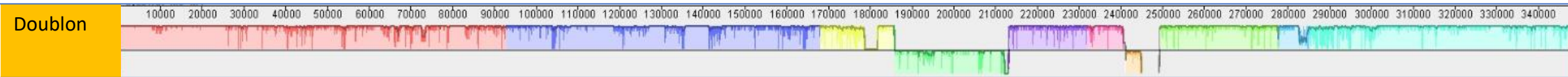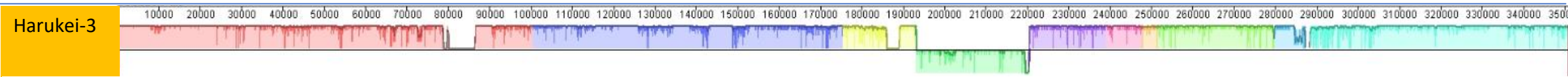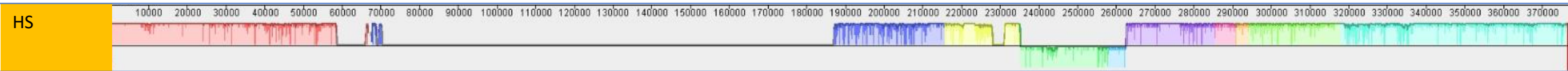

Supplement: Supplementary file 3 — Figure S3 Microsynteny analysis of M5-M4 sequences spanning Vat obtained from seven melon lines. A/ Self dot plots [41]. B/ Mauve alignment [39] of the M5-M4 regions in reference to the DHL92 M5-M4 sequence. Local collinear blocks are represented by blocks of the same color connected by lines. [file 41438_2021_507_MOESM3_ESM.pdf]
