# Supplementary material for: Building a cluster of NLR genes conferring resistance to pests and pathogens: the story of the Vat gene cluster in cucurbits
Source: Hortic Res. 2021 Apr 1;8:72. doi: 10.1038/s41438-021-00507-0 (PMC8012345; doi:10.1038/s41438-021-00507-0)

A/ amplicons obtained with Z649FR primers

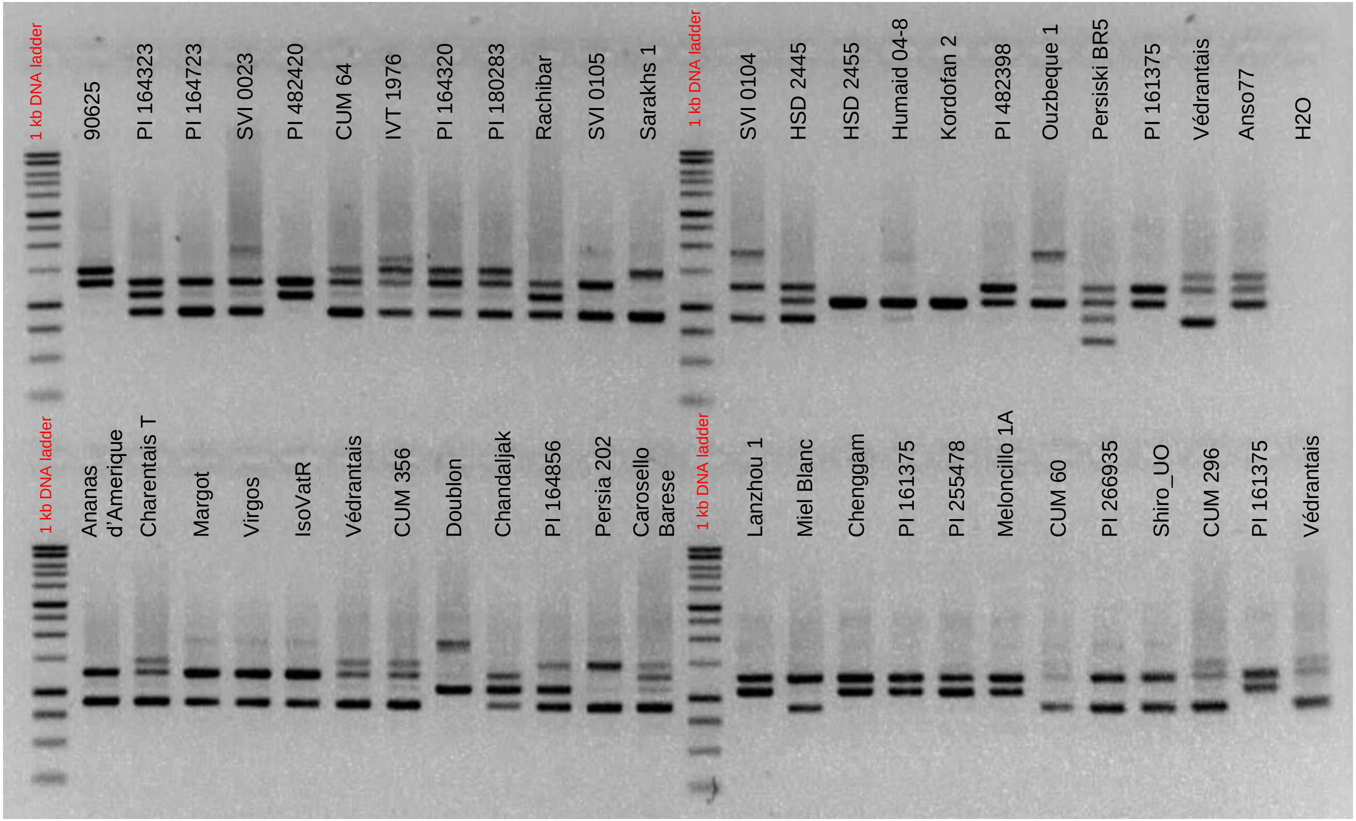

A/ amplicons obtained with Z649FR primers

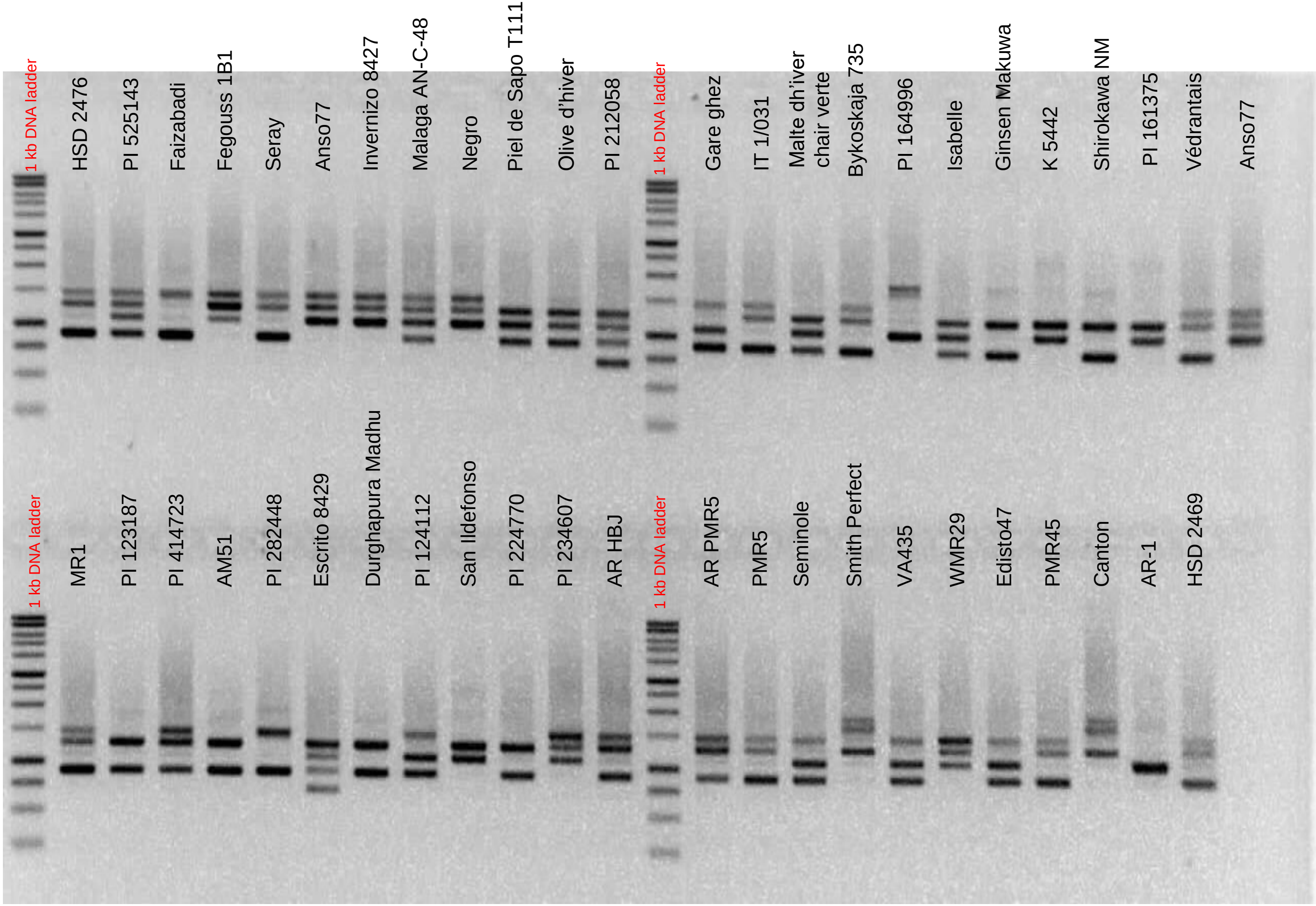

B/ amplicons obtained with Z6097F/Z6095R primers (*Vatx* with one R65aa)

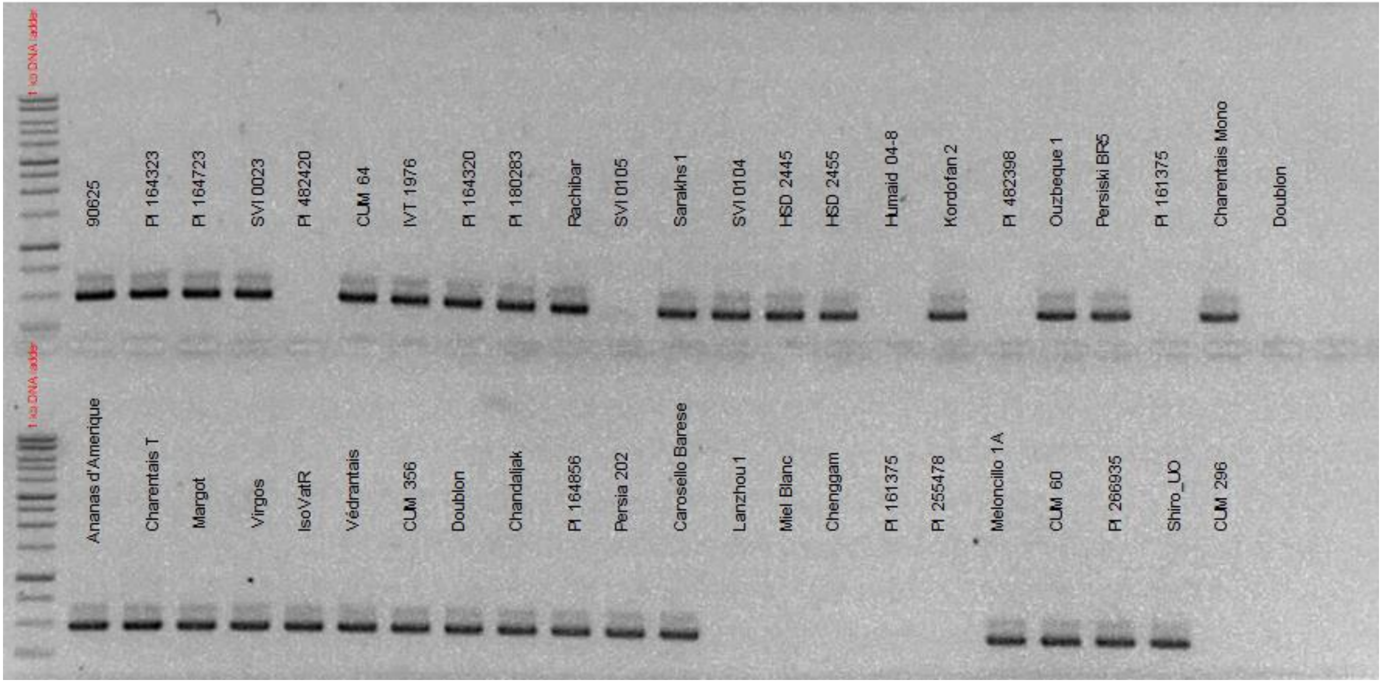

B/ amplicons obtained with Z6097F/Z6095R primers (*Vatx* with one R65aa)

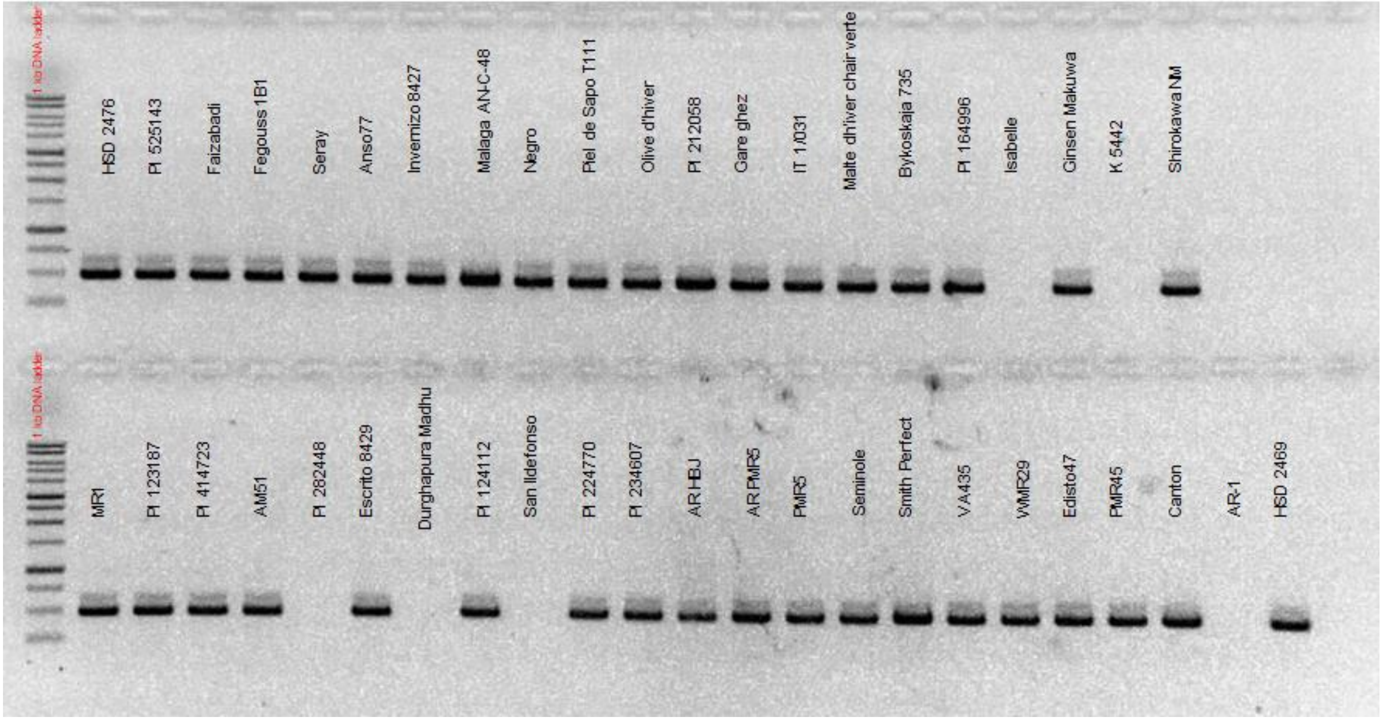

C/ amplicons obtained with Z5474FR primers (*VatRev*)

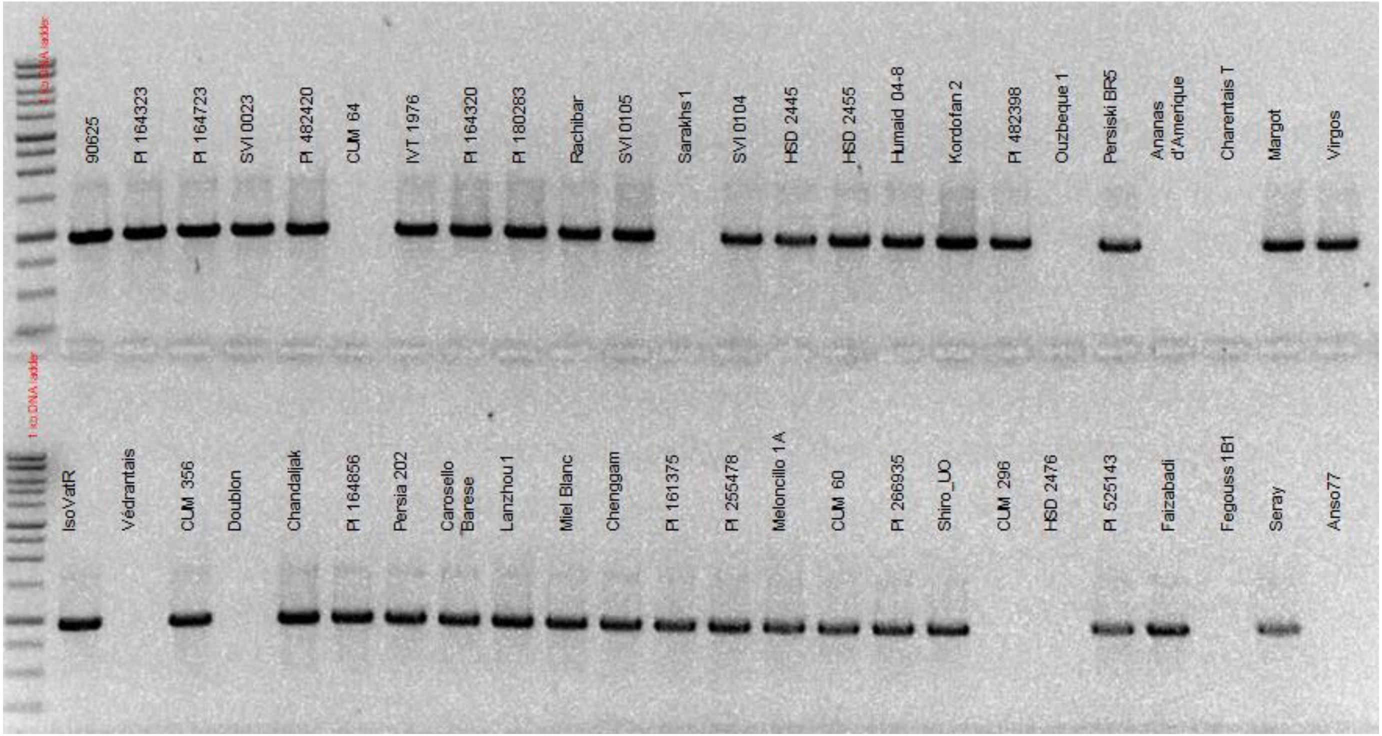

C/ amplicons obtained with Z5474FR primers (*VatRev*)

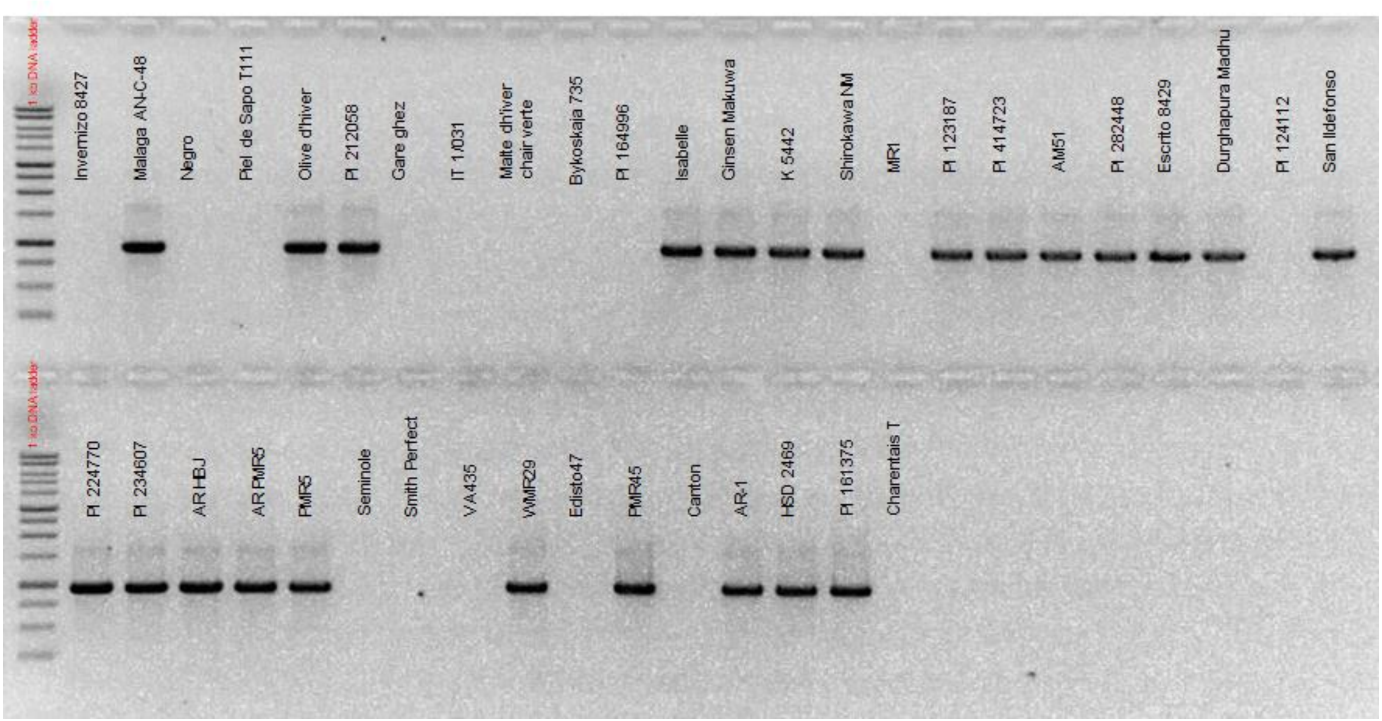

Supplement: Supplementary file 4 — Figure S4 Gel picture obtained from electrophoresis of Vat-homolog amplicons. A/ The amplicons were obtained by PCR using Z649 F and R primers located in the introns on each side of the exon 2 that spans the R65aa motifs in the LRR2 part of Vat. Band numbers observed for each line corresponded to a minimal number of Vat-homologs in the line; band sizes allowed to infer the number of R65aa motifs in these Vat-homologs. B/ The amplicons were obtained by PCR using Z6097F and Z6095R primers located at the beginning and the end of exon 2; they were specific to Vatx with one R65aa, and produced amplicons with 467 bp.C/ The amplicons were obtained by PCR using Z5474 F and R primers located at the end of exon 1 of VatRev; they produced amplicons with 933 bp when VatRev was present. [file 41438_2021_507_MOESM4_ESM.pdf]
