# Supplementary material for: Building a cluster of NLR genes conferring resistance to pests and pathogens: the story of the Vat gene cluster in cucurbits
Source: Hortic Res. 2021 Apr 1;8:72. doi: 10.1038/s41438-021-00507-0 (PMC8012345; doi:10.1038/s41438-021-00507-0)
Supplement: Supplementary file 5 — Table S1 Manual annotation of Vat-homologs sequences located in the M5-M4 region for seven melon genotypes. The Vat-1 gene from PI 161375 was used as a reference sequence to find the set of Vat-related sequences by BLAST. Positions of the different Vat-homologs and Pseudo-Vat are indicated relative to M5. The transposable elements present in the insertions of PseudoX_Vat have been annotated after BLASTX on NCBI. [file 41438_2021_507_MOESM5_ESM.pdf]

Melon line	M5_M4	*Vat* -related in M5_M4 region			CDS						Protein
	Positions	Names	Start	Size	Exon	Start from M5	End from M5	Size	# R65aa	% ident to PI_Vat1	Size (aa)
**PI 161375, Contig 1-610 316 bp**	114 475..416 005	**PI_Vat1**	58 854	5 896	1	58 854	61 202	2 349		-	1467
					2	61 775	62 908	1 134	4	-	
					3	63 730	64 587	858		-	
					4	64 687	64 749	63		-	
		**PI_Vat2**	81 426	5 725	1	81 426	83 774	2 349		98	1404
					2	84 347	85 285	939	3	-	
					3	86 125	86 988	864		92	
					4	87 088	87 150	63		98	
		**PI_pseudo1_Vat**	100 244	12 940	1	100 244	102 552	2 309		89	STOP
					2 (1)	103 436	?		1?	-	
					3	112 165	113 047	883		85	
					4	113 124	113 183	60		78	
		**PI_VatRev**	237 855	5 247	4	237 855	237 908	54		89	1380
					3	237 978	238 832	855	1	83	
					2	239 230	239 775	546		-	
					1	240 231	243 101	2 871		75	

(1) insertion of 8.5 kb beetween exon2 and exon3 containing a LINE-1 retrotransposable element ORF2 protein

(BLASTX on NCBI: 346/576 aa on KAA0041367.1) 148/284 aa on KAA0046834.1) and a CACTA en-spm transposon protein (BLASTX on NCBI: 148/284 aa on KAA0046834.1)

Melon line	M5_M4	*Vat* -related in M5_M4 region			CDS						Protein
	Positions	Names	Start	Size	Exon	Start from M5	End from M5	Size	# R65aa	% ident to PI_Vat1	Size (aa)
**DOUBLON, Contig 331 - 6 870 815 bp**	3 894 754..4 242 284	**DB_Vat1**	60 142	5 042	1	60 142	62 496	2355		97	1405
					2	63 042	63 980	939	3	-	
					3	64 164	65 021	858		94	
					4	65 121	65 183	63		98	
		**DB_Vat2**	77 475	6 404	1	77 475	79 823	2349		98	1665
					2	80 396	82 114	1719	7	-	
					3	82 853	83 716	864		95	
					4	83 816	83 878	63		98	
		**DB_pseudo1_Vat**	96 416	3 304	1	96 416	99 188	2773		89	STOP
					2 (2)	99 612	99 719	108	1?	-	
		**DB_Vat3**	108 072	11 496	1	108 072	110 924	2853		91	1436
					2	111 385	111 930	546	1	-	
					3	118 583	119 431	849		87	
					4	119 508	119 567	60		78	
		**DB_pseudo_VatRev**	274 198	10 183	4	274 198	274 251	54		75	STOP
					3	274 324	275 178	855		83	
					2	275 576	276 125	550	1	-	
					1 (3)	276 581	284 380	7800		89	

(2) DB_pseudo1_Vat: the end of the gene was missing (exon2-exon3-exon4),
and the donwstream 2.54 kb contained a LINE-1 retrotransposable element ORF2 protein (BLASTX on NCBI: 429/795 aa on KAA0039770.1)

(3) DB_pseudo_VatRev: insertion of 4.9 kb in exon1 containing an integrase (BLASTX on NCBI: 1352/1360 aa on TYK16225.1)

Melon line	M5_M4	*Vat* -related in M5_M4 region			CDS						Protein
	Positions	Names	Start	Size	Exon	Start from M5	End from M5	Size	# R65aa	% ident to PI_Vat1	Size (aa)
**ANSO77 , Contig 104 - 6 987 109 bp**	4019 113..434 2617	**AN_Vat1**	59 723	5 825	1	59 723	62 077	2 355		98	1471
					2	62 650	63 783	1 134	4	95	
					3	64 522	65 385	864		93	
					4	65 485	65 547	63		92	
		**AN_Vat2**	83 698	5 704	1	83 698	86 052	2 355		99	1406
					2	86 598	87 536	939	3	-	
					3	88 376	89 239	864		97	
					4	89 339	89 401	63		97	
		**AN_Vat3**	105 527	6 071	1	105 527	107 875	2 349		96	1532
					2	108 426	109 754	1 329	5	-	
					3	110 578	111 435	858		97	
					4	111 535	111 597	63		92	
		**AN_Vat4**	132 478	5 725	1	132 478	134 826	2 349		98	1404
					2	135 399	136 337	939	3	-	
					3	137 177	138 040	864		92	
					4	138 140	138 202	63		98	
		**AN_pseudo1_Vat**	151 247	3 272	1	151 247	153 523	2 277		87	STOP
					2 (4)	154 408	154 518	111	1?	-	
		**AN_Vat5**	162 922	5 268	1	162 922	165 774	2 853		91	1441
					2	166 235	166 780	546	1	-	
					3	167 187	168 053	867		83	
					4	168 130	168 189	60		78	
		**AN_pseudo_VatRev**	250 237	10 115	4	250 237	250 290	54		75	STOP
					3	250 360	251 216	857		78	
					2	251 614	252 159	546	1	-	
					1 (5)	252 615	260 351	7 737		89	

(4) AN_pseudo1_Vat: the end of the gene was missing (exon2-exon3-exon4) and the donwstream 2.54 kb contained a LINE-1 retrotransposable element ORF2 protein (BLASTX on NCBI: 346/576 aa on KAA0041367.1)

(5) AN_pseudo_VatRev: insertion of 5 Kb in exon1 containing an integrase (BLASTX on NCBI: 1299/1932 aa on TYK16225.1)

**Melon line**	**M5_M4**	***Vat*-related in M5_M4 region**			**CDS**						**Protein**
	**Positions**	**Names**	**Start**	**Size**	**Exon**	**Start from M5**	**End from M5**	**Size**	**# R65aa**	**% ident to PI_Vat1**	Size (aa)
**DHL92, Chr05 - 29 075 199 bp**	26 085 380..26 434 884	**DH_Vat1**	60 146	5 042	1	60 146	62 500	2 355		97	1404
					2	63 046	63 984	939	3	-	
					3	64 168	65 025	858		94	
					4	65 125	65 187	63		98	
		**DH_Vat2**	77 482	4 852	1	77 482	79 836	2 355		97	1339
					2	80 387	81 130	744	2	-	
					3	81 314	82 171	858		93	
					4	82 271	82 333	63		98	
		**DH_pseudo1_Vat**	104 783	5 478	1	104 783	107 100	2 318		88	STOP
					2	107 973	108 494	522	1	-	
					3	109 249	110 090	842		82	
					4	110 198	110 260	63		80	
		**DH_Vat3**	118 631	6 412	1	118 631	120 967	2 337		93	1462
					2	121 524	122 654	1 131	4	-	
					3	123 394	124 251	858		91	
					4	124 980	125 042	63		95	
		**DH_Vat4**	140 318	6 476	1	140 318	142 666	2 349		97	1662
					2	143 239	144 957	1 719	7	-	
					3	145 774	146 631	858		94	
					4	146 731	146 793	63		95	
		**DH_pseudo2_Vat**	176 341	3 283	1	176 341	179 158	2 818		88	STOP
					2 (6)	179 540	179 623	84	1?	-	
		**DH_pseudo3_Vat**	188 591	8 073	1 (7)	188 591	194 248	5 658		90	697
					2	194 709	195 254	546	1	-	
					3	195 645	196 527	883		83	
					4	196 604	196 663	60		78	
		**DH_pseudo_VatRev**	278 747	11 909	4	278 747	278 800	54		75	
					3	278 870	279 726	857		77	
					2	280 124	280 669	546	1	-	
					1 (8)	281 125	290 655	9 531		89	STOP

(6) DH_pseudo2_Vat: the end of the gene was missing (exon2-exon3-exon4) and the donwstream 2.54 kb contained a LINE-1 retrotransposable element ORF2 protein (BLASTX on NCBI: 384/720 aa on TYK06777.1)

(7) DH_pseudo3_Vat: insertion of 2.78 kb in exon1 containing a LINE-1 retrotransposable element (BLASTX on NCBI: 808/893 aa on KAA0046762.1)

(8) DH_pseudo_VatRev: insertion of 6.65 kb in exon1 containing a transposase (BLASTX on NCBI: 608/779 aa on KAA0064110.1)

Melon line	M5_M4	*Vat* -related in M5_M4 region			CDS						Protein
	Positions	Names	Start	Size	Exon	Start from M5	End from M5	Size	# R65aa	% ident to PI_Vat1	Size (aa)
**PAYZAWAT, Chr05- 29 138 414 bp**	883 876..1 221 532	**PZ_Vat1**	60 146	6 404	1	60 146	62 494	2 349		98	1664
					2	63 067	64 785	1 719	7	-	
					3	65 524	66 387	864		94	
					4	66 487	66 549	63		98	
		**PZ_Vat2**	78 221	4 852	1	78 221	80 575	2 355		97	1339
					2	81 126	81 869	744	2	-	
					3	82 053	82 910	858		93	
					4	83 010	83 072	63		97	
		**PZ_pseudo1_Vat**	96 146	3 312	1	96 146	98 462	2 317		89	STOP
					2 (9)	99 345	99 457	113	1?	-	
		**PZ_Vat3**	107 801	11 495	1 (10)	107 801	110 653	2 853		91	1435
					2	111 114	111 659	546	1	-	
					3	118 311	119 159	849		87	
					4	119 236	119 295	60		78	
		**PZ_VatRev**	268 750	5 246	4	271 125	273 995	2 871		75	1380
					3	270 124	270 669	546		78	
					2	268 873	269 727	855	1	-	
					1	268 750	268 803	54		89	

(9) PZ_pseudo1_Vat: the end of the gene was missing (exon2-exon3-exon4) and
the donwstream 2.54 kb contained a LINE-1 retrotransposable element ORF2 protein(BLASTX on NCBI: 393/790 aa on TYK06777.1)
(10) PZ_Vat3: insertion in intron2 (6.61 kb) containing a LINE-1 retrotransposable element (BLASTX on NCBI: 1779/1804 aa on KAA0057507.1)

Melon line	M5_M4	*Vat* -related in M5_M4 region			CDS						Protein
	Positions	Names	Start	Size	Exon	Start from M5	End from M5	Size	# R65aa	% ident to PI_Vat1	Size (aa)
**HARUKEI-3, chr05 - 29 724 715 bp**	26 694 405..27 045 164	**HRK_Vat1**	60 138	5 042	1	60138	62492	2 355		97	1 404
					2	63038	63976	939	3	-	
					3	64160	65017	858		94	
					4	65117	65179	63		98	
		**HRK_pseudo1_Vat**	77 473	13 887	1 (11)	77473	87304	9 832		-	STOP
					2	87877	89595	1 719	7	-	
					3	90334	91197	864		95	
					4	91297	91359	63		98	
		**HRK_pseudo2_Vat**	103 898	2 317	1 (12)	103898	106214	2317	-	89	
		**HRK_Vat2**	115 515	11 360	1	115515	118367	2 853		91	1 141
					2	118828	119373	546	1	-	
					3	126010	126874	865		87	
					4	126951	127010	60		73	
		**HRK_pseudo_VatRev**	276 211	10 184	4	276211	276264	54		70	
					3	276337	277191	855		84	
					2	277589	278139	551	1	-	
					1 (13)	278595	286394	7 800		90	STOP

(11) HRK_pseudo1_Vat: insertion of 7.49 kb in exon1. No similarity found with transposable elements

(12) HRK_pseudo2_Vat: the end of the gene was missing (exon2-exon3-exon4) and

the donwstream 2.54 kb contained a LINE-1 retrotransposable element ORF2 protein (BLASTX on NCBI: 348/577 aa on KAA0041367.1)

(13) HRK_pseudo_VatRev: insertion of 5.14 kb in exon1 containing an integrase (BLASTX on NCBI: 1355/1360 aa o TYK16225.1)

Melon line	M5_M4	*Vat* -related in M5_M4 region			CDS						Protein
	Positions	Names	Start	Size	Exon	Start from M5	End from M5	Size	# R65aa	% ident to PI_Vat1	Size (aa)
**HS**, Chr05 - 29 735 421 bp	26 775 630..27 152 309	**HS_Vat1**	70 910	6 088	1	70910	73246	2 337		93	1 530
					2	73803	75131	1329	5	-	
					3	75972	76835	864		94	
					4	76935	76997	63		98	
		**HS_pseudo1_Vat**	93 288	5 714	1	93288	95629	2 342		98	STOP
					2	96202	97137	936	3	-	
					3	97976	98841	864		92	
					4	121002	121064	63		97	
		**HS_pseudo2_Vat**	115 338	5 727	1	115338	117688	2351		98	STOP
					2	118261	119198	938	3	-	
					2	120039	120902	864		92	
					1	121002	121064	63		98	
		**HS_Vat2**	137 421	5 727	1	137421	139769	2 349		98	1 404
					2	140343	141281	939	3	-	
					3	142122	142985	864		92	
					4	143085	143147	63		98	
		**HS_Vat3**	159 453	5 726	1	159453	161801	2 349		98	1 176
					2	162373	163311	939	3	-	
					3	164152	165016	865		92	
					4	165116	165178	63		98	
		**HS_pseudo3_Vat**	181 498	7 134	1	181498	183845	2 348		98	STOP
					2 (14)	-	-		-	-	
					3	187769	188631	863		83	
					4	188706	188765	60		73	
		**HS_VatRev**	312 995	5 247	4	312995	313048	54		89	1380
					3	313118	313972	855	1	83	
					2	314370	314915	546		-	
					1	315371	318241	2 871		75	

(14) HS_pseudo3_Vat: exon2 was missing and replaced by 3.92 kb between exon1 and exon3. No similarity found with transposable elements
