# Supplementary material for: Building a cluster of NLR genes conferring resistance to pests and pathogens: the story of the Vat gene cluster in cucurbits
Source: Hortic Res. 2021 Apr 1;8:72. doi: 10.1038/s41438-021-00507-0 (PMC8012345; doi:10.1038/s41438-021-00507-0)
Supplement: Supplementary file 6 — Table S2 Primers used for RT-PCR and for CDS sequencing of the different Vat-homologs in five melon lines and two other Cucurbits. F: forward - R: reverse. In bold: primer pairs for RT-PCR. In italic: (1) PI_Vat-1 specific primer; (2) PI_Vat-2 specific primer [file 41438_2021_507_MOESM6_ESM.pdf]

Genotype	*Vat* -homolog	Full CDS expected size	Primer Name	Primer sequence (5'-3')	Localization	Size of sequenced CDS
***Cucumis melo* PI161375**	***PI_Vat1*** (4 R65aa) and ***PI_Vat2*** (3 R65aa)		**Z717F**	**F: CTCTGCACTGTCTCTTCTCCATTTC**	**5'UTR**	
			Z761R	R: GGCGCACATTTGATTTGAGAT	exon1	
		4404	Z762F	F: GAAGCCTTTTGATGAGGTGGTAA	exon1	4404
			Z762R	R: AGTTTCTACCACCGACACACG	exon1	
			Z763F	F: TGATATGGTTCGTGATGTAGCC	exon1	
			Z764F	F: GGGAAGGAGAAGAATGGTATGAAG	exon1	
			Z1431F	F: ATGCAAAGAGTTTGAAGATG	exon2	
			LRR915F	F: GTTGGAATCAAAGCAATGGGA	exon2	
		4215	*Z1431R (1)*	*R: GAAGATTACATAACATCAACGA*	*exon2*	4215
			Z1617R (2)	*R: GAAGATTATATAATTCCAACCTAA*	*exon2*	
			LRR915R	R: AACAACTTAGAACCATCTCCCAGC	exon3	
			**Z717R**	**R: CAAGATTCTGACCTTTTCCTTGTGG**	**3'UTR**	
	PI_VatRev (1 R65aa)	4143	**Z5469F**	**F: GGTTTCAAGAATCTGACCTTCC**	**3'UTR**	2374
			Z5471F	F: TTGAAGCGATGGAAGGATTC	exon3	
			Z5473F	F: TTTCCTTGAGTTTGGAAAAGGA	exon2	
			Z5473R	R: TTGCATGTTGGATGACGTTT	exon1	
			**Z5474R**	**R: CGTTTACGGGGATGTGATTT**	**exon1**	
			**Z5474F**	**F: ATGTGGTTGGTTGCCTTCTC**	**exon1**	2702
			Z5475F	F: CAAAAGTTCCTAAACGTAATTCCTC	exon1	
			Z5476F	F: TTTCAACTCACTTCCTTCAACAATT	exon1	
			Z5475R	R: CTCAACTCAAATGTGCGCC	exon1	
			**Z5469R**	**R: ATGATTATGGTTCATTCTTTTTGGG**	**5'UTR**	
	DB_Vat1 (3 R65aa)	4215	**Z5896F**	**F: GAGCCAATTGACACAGCTGAAAGTT**	**exon1**	1638
			Z5899F	F: CTTTTGCATACCAGAACAAGAACAGAAT	exon1	
			Z5896R	R: GCTCCATATCATCTCCAAACTCTC	exon2	
			**Z5899R**	**R: GATAGAATCAAACTCTGTAAATCCATAG**	**exon3**	
			**Z717F**	**F: CTCTGCACTGTCTCTTCTCCATTTC**	**5'UTR**	
			Z761R	R: GGCGCACATTTGATTTGAGAT	exon1	
			Z762F	F: GAAGCCTTTTGATGAGGTGGTAA	exon1	

***Cucumis melo* Doublon**	***DB_Vat2*** (7 R65aa)	4995	Z762R	R: AGTTTCTACCACCGACACACG	exon1	3243
			Z763F	F: TGATATGGTTCGTGATGTAGCC	exon1	
			Z763R	F: AAGCACCTTCCTGATTCCATC	exon1	
			Z764F	F: GGGAAGGAGAAGAATGGTATGAAG	exon1	
			Z764R	R: GGCACGTTATTGCTCCATATCA	exon2	
			Z1431F	F: ATGCAAAGAGTTTGAAGATG	exon2	
			**Z5900R**	**R: GGTAGTTTAGATAGAGTCAATCTCCT**	**exon3**	
			**Z5900F**	**F: CTTTTGCATACAAGAACAGAATCAAAAAC**	**exon1**	1328
			Z5902F	F: TCAAGCCACTTGAAGATGTAAGCA	exon2	
			LRR915R	R: AACAACTTAGAACCATCTCCCAGC	exon3	
			**Z5904R**	**R: ATTATCCTCCCAATATTCTCTGGTG**	**exon3**	
	DB_Vat3 (1 R65aa)	4308	**Z5895F**	**F: GCCAATTGACACAACTCAAAGTGTTA**	**exon1**	1744
			Z5898F	F: TTTATGAAAACAACACCAGCTTCATTGG	exon1	
			**Z5898R**	**R: GGTAGTTTAGATAGAGCAAATCTCCG**	**exon3**	
***Cucumis melo* Anso77**	***AN_Vat1*** (4 R65aa)	4416	**Z5946F**	**F: CCAAAGCAAATTAAAGTATACTTTGCCA**	**exon1**	551
			Z5946R	R: TAGAAGCTCACAGGGATTTTTGCTCCACAG	exon2	
			Z5947F	F: CTGTGGAGCAAAAATCCCTGTGAGCTTCTA	exon2	627
			**Z5947R**	**R: CTTTTGCATTCTTCTACTGTCAAACCTTC**	**exon3**	
	AN_Vat2 (3 R65aa)	4221	**Z5948F**	**F: CCAAAGCAAATTAAAGTATACTTTTGCT**	**exon1**	1500
			**Z5948R**	**R: CTTTTGCATTCTTCTATTGTCAAAGACTG**	**exon3**	
	AN_Vat3 (5 R65aa)	4599	**Z5949F**	**F: ATCGGGTAATCATACAACAGTGTCT**	**exon1**	680
			Z764F	F: GGGAAGGAGAAGAATGGTATGAAG	exon1	
			Z5949R	R: GTATTCTCTTCTAAGTCTTGGACATTTA	exon2	813
			**Z5950R**	**R: CTTTTGCATTCGTGTATTCTCAAATGTTC**	**exon3**	
	AN_Vat4 (3 R65aa)	4215	**Z5951F**	**F: CAAATTAAAGTATACTTTTGGACCCACA**	**exon1**	1465
			**Z5951R**	**R: CTTTTGCATTCATTTATTGTCAAATCTTG**	**exon3**	
	AN_Vat5 (1 R65aa)	4326	**Z5952F**	**F: TTTAGCTGGGGAGTTGAATCTTG**	**exon1**	1564
			Z5953F	F: GAAGATATATTTATGAAAACAACACCAG	exon1	
			Z5952R	R: ATCTTCAAATTCGTTGCGCTCCT	exon2	
			**Z5953R**	**R: ATTCTCAAATGTTCAAGATGCACAAGTT**	**exon3**	

Cucumis melo **Piel de sapo (DHL92)**	***DH_Vat1*** (3R65aa)	4215	**Z6099F**	**F: CATCTCAGAACGATCACATACGTAT**	**exon1**	1374
			Z764F	F: GGGAAGGAGAAGAATGGTATGAAG	exon1	
			**Z6098R**	**R: CCACAATCTGAAATTCCTAGAGAACA**	**exon3**	
	DH_Vat2 (2 R65aa)	4020	**Z6100F**	**F: ACTGTAGCATTGACGTGGAAGG**	**exon1**	1011
			Z764F	GGGAAGGAGAAGAATGGTATGAAG	exon1	
			Z1431F	ATGCAAAGAGTTTGAAGATG	exon2	
			**Z857R**	**CCATTGGTAAATGGGTTGAGTT**	**exon3**	
	DH_Vat3 (4 R65aa)	4389	**Z6104F**	**F: TTGCCAATCAATGTATACTTTTGGAC**	**exon1**	1517
			LRR915R	R: AACAACTTAGAACCATCTCCCAGC	exon3	
			**Z6129R**	**CCACCACATTCGGAAATGGA**	**exon3**	
	DH_Vat4 (7 R65aa)	4989	**Z6106F**	**F: AGCAAATTAAAGTATACTTTTGAATCCAC**	**exon1**	600
			**Z6106R**	**R: TTTCTTCGATAGATGCTCCTACTAATA**	**exon3**	847
Cucumis melo **Payzawat**	***PZ_Vat1*** (7 R65aa)	4995	**Z6089F**	**F: CTTGTTCTTAGTTGGGAAGTTGAAG**	**exon1**	764
			**Z6089R**	**R: CTATCACATTTATAAACCGCAAGACT**	**exon3**	911
	PZ_Vat2 (2 R65aa)	4020	**Z6092F**	**F: ACAGCTGAAAGTTTTGAATTTATCTTT**	**exon1**	1418
			**Z6092R**	**R: CCACATTCAGAAATGGATACATCAG**	**exon3**	
	PZ_Vat3 (1 R65aa)	4308	**Z6096F**	**F: GTTGCCAACGTGACGGAAG**	**exon1**	1500
			Z6095R	R: CTTTTTCTTCCATATAACCTCCACA	exon2	
			**Z6096R**	**R: CATCACACTTTATCACATCAAAAACTAC**	**exon3**	
	PZ_VatRev (1 R65aa)	4326	**Z5471F**	**F: TTGAAGCGATGGAAGGATTC**	**exon3**	502
			**Z5471R**	**R: GGGAAGAAAAAGTTGGCAGA**	**exon2**	
			**Z5473F**	**F: TTTCCTTGAGTTTGGAAAAGGA**	**exon2**	1110
			**Z5474R**	**R: CGTTTACGGGGATGTGATTT**	**exon1**	
Cucumis sativus **cv 9930**	***CS9930_Vat-related***	5595	**Z6041F**	**F: TGGTTCATTCGTTGTGGGTTTTTAGC**	**5'-UTR**	5020
			**Z6041R**	**R: TGTTTGCCATACCCACATCAACACT**	**exon1**	
			**Z6042F**	**F: TGGAATTTATTTAAGGCAATGGCGGGT**	**exon1**	
			Z6042R	R: TCCTTCCTTCGTACCATTCTTCCCC	exon1	
			Z6043F	F: CGCCACAAGCTCTTTACTCATTTGC	exon1	
			Z6043R	R: GCATGAAGCAATCTCTACTTCCTCCAG	exon2	
			Z6044F	F: AAACGAGGAAGCAACCAACCACA	exon1	
			Z6044R	R: TGCACAAGGGTTGTAGCCACCAAA	exon3	
			Z6046F	F: ACCCAAGCTTAGGCATTTGTGGAGT	exon3	

			Z6046R	R:TCCTTCCCAAGAGCCACTGTCA	exon4	
			**Z6045R**	**R: GCACGAGCGTTGTAGCTACCGAT**	**exon5**	
	Cs9930_Vat-outofcluster	4119	**Z6075F**	**F: AGGAAGGGCACCTATTCTACGAAAGA**	**exon1**	982
			**Z6075R**	**R: TGCTCCCTAATTCACATCCCCGT**	**exon1**	
			**Z6078F**	**F: ACAAGTCCTATCGCCCTCCAAACT**	**exon2**	839
			**Z6078R**	**R: AGCTTGTTAGGTTGGAACAAGAAGTAATGA**	**exon3**	
***Cucumis sativus* cv Gy14**	***CsGy14_Vat-related***	5595	**Z6041F**	**F: TGGTTCATTCGTTGTGGGTTTTTAGC**	**5'-UTR**	5054
			**Z6041R**	**R: TGTTTGCCATACCCACATCAACACT**	**exon1**	
			**Z6042F**	**F: TGGAATTTATTTAAGGCAATGGCGGGT**	**exon1**	
			Z6042R	R: TCCTTCCTTCGTACCATTCTTCCCC	exon1	
			Z6043F	F: CGCCACAAGCTCTTTACTCATTTGC	exon1	
			Z6043R	R: GCATGAAGCAATCTCTACTTCCTCCAG	exon2	
			Z6044F	F: AAACGAGGAAGCAACCAACCACA	exon1	
			Z6044R	R: TGCACAAGGGTTGTAGCCACCAAA	exon3	
			Z6046F	F: ACCCAAGCTTAGGCATTTGTGGAGT	exon3	
			Z6046R	R:TCCTTCCCAAGAGCCACTGTCA	exon4	
			**Z6045R**	**R: GCACGAGCGTTGTAGCTACCGAT**	**exon5**	
	CsGy14_Vat-outofcluster	4119	**Z6075F**	**F: AGGAAGGGCACCTATTCTACGAAAGA**	**exon1**	971
			**Z6075R**	**R: TGCTCCCTAATTCACATCCCCGT**	**exon1**	
			**Z6079F**	**F: AGGCATTTGGGGAGTGAATGCTC**	**exon3**	985
			**Z6079R**	**R: GGTGTCCATCAGAGTGCATCTTGG**	**3'-UTR**	

***Citrullus lanatus* cv Charleston Gray**	***Cla_Vat-related***	4264	**Z6058F**<br>Z6131F<br>Z6059F<br>Z6058R<br>Z6060F<br>Z6059R<br>Z6060R<br>**Z6061R**	**F: CCATTCGTTGTGGGTCTTTT**<br>F: AGGAGGAAGGGCACTGAGGCT<br>F: TATTCAAGGCAATGGCAGGT<br>R: CGAATACACTGCCTTGGTCA<br>F: GTCGAAATTCCTGCAACC AT<br>R: AAGCTGTGGCAAGTACCTGAG<br>R: TGTCAAATTTCGGAGCACAA<br>**R: CTTCAGCTCCTCCCTCAATG**	**5'UTR**<br>exon1<br>exon1<br>exon1<br>exon1<br>exon1<br>exon 2<br>**exon 3**	3821
