# Supplementary material for: Building a cluster of NLR genes conferring resistance to pests and pathogens: the story of the Vat gene cluster in cucurbits
Source: Hortic Res. 2021 Apr 1;8:72. doi: 10.1038/s41438-021-00507-0 (PMC8012345; doi:10.1038/s41438-021-00507-0)
Supplement: Supplementary file 8 — Table S4 Primers used for long-range PCR and genomic DNA sequencing of the different Vat homologs in melon lines. F: forward, R: reverse. [file 41438_2021_507_MOESM8_ESM.pdf]

Marker Name	Primer sequence (5’-3’)	Localization
Z717	F : CTCTGCACTGTCTCTTCTCCATTTC	5'UTR
	R : CAAGATTCTGACCTTTTCCTTGTGG	3'UTR
Z761	R : GGCGCACATTTGATTTGAGAT	exon1
Z762	F: GAAGCCTTTTGATGAGGTGGTAA	exon1
	R : AGTTTCTACCACCGACACACG	exon1
Z763	F : TGATATGGTTCGTGATGTAGCC	exon1
	R : AAGCACCTTCCTGATTCCATC	exon1
Z764	F : GGGAAGGAGAAGAATGGTATGAAG	exon1
	R : GGCACGTTATTGCTCCATATCA	exon2
Z649	F : TGTCACAAACTGAACTTTTAAGGA	intron1
	R : CTGTTCAACTAACAGAACCAATTC	intron2
LRR915	F : GTTGGAATCAAAGCAATGGGA	exon2
	R : AACAACTTAGAACCATCTCCCAGC	exon3
Z765F	F : TCCAAAATGTAATTCAAAAACACC	intron2
V632	F : CCCAGCAACATACTGATTCCAAGC	exon3
	R : CTGGTGATGACATTCATATCTTCC	exon3
Z5895	F: GCCAATTGACACAACTCAAAGTGTTA<br>R : CATATCATCTTCAAATTCGTTGCGC	exon1<br>exon2
Z5474	F: ATGTGGTTGGTTGCCTTCTC<br>R : CGTTTACGGGGATGTGATTT	exon1<br>exon1
Z6097F<br>Z6095R	F : TGAAAATTAGGAGCGCAACGAA<br>R : CTTTTTCTTCCATATAACCTCCACA	exon2<br>exon2
