# Supplementary material for: Building a cluster of NLR genes conferring resistance to pests and pathogens: the story of the Vat gene cluster in cucurbits
Source: Hortic Res. 2021 Apr 1;8:72. doi: 10.1038/s41438-021-00507-0 (PMC8012345; doi:10.1038/s41438-021-00507-0)
Supplement: Supplementary file 9 — Methods S1 Genomic resource production for PI 161375, Anso77 and Doublon and de novo genome assemblies for Anso77 and Doublon. High molecular weight (HMW) genomic DNA production and NGS sequencing with PacBio (for PI 161375 and Doublon) and Oxford Nanopore Technologies (for Anso 77 and Doublon). The genome assemblies were performed using canu v.1.8 (for Anso77 and Doublon). [file 41438_2021_507_MOESM9_ESM.pdf]

Genomic resource production for PI 161375, Anso77 and Doublon

High molecular weight (HMW) genomic DNA was produced by INRAe-CNRGV for sequencing of large genomic regions spanning *Vat* in the three melon lines. Plants were grown in a greenhouse and transferred in a dark room 3 to 5 days before collecting the youngest leaves. HMW DNAs were purified from one gram of very young frozen leaves according to the Bionano Prep Plant tissue DNA Isolation Liquid Nitrogen Grinding Protocol (30177 - Bionano Genomics) with the following specifications and modifications. Briefly, the leaves were broken in liquid nitrogen and then disrupted with a rotor stator in an homogenization buffer. Nuclei were washed and then embedded in agarose plugs. After overnight proteinase K digestion in the presence of Lysis Buffer (Bionano Genomics) and one-hour treatment with RNAse A (Qiagen), plugs were washed four times in 1x Wash Buffer (Bionano Genomics) and five times in 1x TE Buffer (ThermoFisher Scientific). Then, plugs were melted two minutes at 70°C and solubilized with 2 µL of 0.5 U/µL AGARase enzyme (ThermoFisher Scientific) for 45 minutes at 43°C. A dialysis step was performed in 1x TE Buffer (ThermoFisher Scientific) for 45 minutes to purify DNA from any residues. The DNA samples were quantified by using the Qubit dsDNA BR Assay (Invitrogen). The presence of megabase size DNA was visualized by pulsed field gel electrophoresis (PFGE).

For PI 161375, we used the BAC library described in [4] representing ~12-fold coverage of the melon genome (144 plates, 55,296 BAC clones and mean insert size of 110 kb); screening and sequencing were performed by INRAe-CNRGV. BAC clones were spotted on nylon membrane, screened with specific radioactive labelled probes designed on specific markers located in a 1 Mb region spanning the *Vat* locus on the melon genome v3.6.1 [10] and revealed by the high-density filter reader program. Positive BAC clones were verified by real-time PCR using specific primers (Additional file 1). Two µg of each individual BAC clone of interest were pooled for construction of a SMRTbell® library using the standard Pacific Biosciences preparation protocol for 10 kb libraries with PacBio® Barcoded Adapters. The pool was sequenced in one SMRTcell using the P6 polymerase and C4 chemistry. Sequencing was performed on a PacBio RS II sequencer by NGI at Upssala (https://ngisweden.scilifelab.se/). After a demultiplexing step, the sequence assembly was performed following the HGAP PacBio workflow [Chin, C.S. et al. Nonhybrid, finished microbial genome assemblies from long-read SMRT sequencing data. *Nature Methods* **10**, 563- (2013)], and using the SMRT® Analysis (V2.3) software suite for HGAP implementation (https://github.com/PacificBiosciences/Bioinformatics-Training/wiki/HGAP). BAC-end sequencing was used for validation of the BAC clone assembly and BAC clone position on the reference melon genome.

Eleven overlapping BAC clones (including BAC3 from [4]) spanned the 1 Mb region surrounding the *Vat* locus represented by three independent contigs with 199 Kb, 610 Kb and 164 Kb sizes. (Additional file 1). The gap sizes between contigs 1 and 2 were 192 and 118 Kb, respectively compared to the genomes of DHL92 and Payzawat, and that between contigs 1 and 3 were 207 and 205 Kb. Four genetic markers (M5, M8, M7 and M4) linked to the *Vat* locus and used for genetic and physical mapping of the *Vat-1* gene [4] were used to validate the BAC clones assembly.

For Anso77 and Doublon, Oxford Nanopore Technologies (ONT) libraries and sequencing were performed by the INRAe_EPGV group using Genoscope facilities (CEA-IbFJ, Evry, France). For Doublon, a sequencing library was prepared from 1,5 µg of HMW genomic DNA (fragment size > 60 Kb measured on an Agilent Technologies 2200 TapeStation) using the 1D gDNA protocol selecting for long reads (SQK-LSK109 ; ONT, Oxford, UK), according to the manufacturer's instructions. 215 ng of the library was loaded on a MinION flow cell (version R9.4.1) and sequenced on a MinION Mk1B sequencing device for 48 h. For Anso77, in order to enhance the recovery of long DNA fragments, the Short Read Eliminator Kit (Circulomics, Baltimore, Maryland, US, Cat #SS-100-101-01) was used starting from 7.6 µg of HMW Genomic DNA according to the Circulomics Short Read Eliminator Kit – Beta Handbook v1.0. A sequencing library was prepared from 1.4 µg Circulomics purified HMW Genomic DNA (fragment size

35-165 Kb measured on an Agilent Technologies Femto Pulse) using the same protocol as that used for the Doublon line. 185 ng of the library was loaded on a MinION Flongle Flow Cell (version R9.4.1) and sequenced on a MinION Mk1B sequencing device for 24 h for library validation, and 925 ng of the same library was loaded on a PromethION Flow Cell (version R9.4.1 revD) and sequenced on a beta PromethION sequencing device for 48 h. The basecalling of the generated raw data (fast5 format) was performed using Guppy Basecalling software (version 2.3.5 for MinION runs; version 3.0.4 for PromethION run) and the FASTQ files were obtained as described in [Istace, B., et al. De novo assembly and population genomic survey of natural yeast isolates with the Oxford Nanopore MinION sequencer. *Gigascience* **6** (2017).]. We obtained 45.1 Gb (N50: 28.1 kb) of raw data for Anso77 and 2.6 Gb (N50: 27.3 kb) for Doublon.

For Anso77, a linked read library was prepared starting with 1 ng of high molecular weight genomic DNA using the Chromium Genome Reagent Kit according to the manufacturer's protocol (https://support.10xgenomics.com/genome-exome/index/doc/user-guide-chromium-genome reagent-kit-v2-chemistry, 10x Genomics, Pleasanton, CA, USA). Sequencing was conducted with an Illumina NovaSeq 6000 with 2 × 150 bp paired-end reads (Illumina, San Diego, CA, USA) according to the manufacturer's protocol, and we retrieved 66 Gb of raw data.

For Doublon, single-molecule real-time long-read sequencing was performed at the Gentyane Sequencing Platform (Clermont-Ferrand, France) with a PacBio Sequel Sequencer (Pacific Biosciences, Menlo Park, CA, USA). The SMRTBell library was prepared using a SMRTbell Express 2 Template prep kit according to the procedure and checklist for preparing gDNA libraries using the SMRTbell Express Template prep kit 2.0. 4 ug of HMW genomic DNA was sheared with the 75 kb program using a Diagenode Megaruptor (Diagenode) generating DNA fragments of approximately 70 kb. A Femto Pulse (Agilent Technologies, Santa Clara, CA, USA) assay was used to assess the fragment size distribution. Sheared genomic DNA were subjected to enzymatic reactions to remove single-strand overhangs and repair any damage potentially present on the DNA backbone. An A-tailing reaction followed by overhang adapter ligation was conducted to generate the SMRTBell template. After 0.45X AMPure PB bead purification, the sample was size-selected using the BluePippin system (Sage Science, Beverly, MA, USA) in order to recover all material above 20 kb. The sample was then purified with 0.45X AMPure PB beads to obtain final libraries of around 75 kb. The SMRTBell library was quality inspected and quantified on a Femto Pulse (Agilent Technologies) and a Qubit fluorimeter with the Qubit dsDNA HS Assay Kit (Life Technologies). A ready-to-sequence polymerase-SMRTBell template complex was created using a Binding Kit 3.0 (PacBio) and V4 primer—the diffusion loading protocol was used according to the manufacturer's instructions. The PacBio Sequel instrument was programmed to load an 8 pM library and the sample was sequenced on four PacBio SMRTCells v2.0 (Pacific Biosciences), while acquiring one 600 min movie per SMRTcell, generating 40.5 Gb (N50: 24.7kb) of raw data.

De novo melon genome assemblies

For Anso77, ONT raw reads were filtered using Nanofilt version 1.1.3 [De Coster, W. et al. (2018) NanoPack: visualizing and processing long-read sequencing data. Bioinformatics **34,** 2666-2669 (2018)] with a quality score of Q > 7 and a length of > 5 kb (36.1 Gb, 95X). The assembly was then performed using canu v.1.8 [Koren, S. et al. Canu: scalable and accurate long-read assembly via adaptive k-mer weighting and repeat separation. *Genome Research* **27**, 722-736 (2017)] with genomeSize=380m, corOutCoverage=40 and minReadLength=1000 settings. A last polishing step with ema v0.6.2 [Shajii, A., Numanagic, I. & Berger, B. Latent variable model for aligning barcoded short-reads improves downstream analyses. In *Research in Computational Molecular Biology, Recomb* Raphael, B.J. ed, pp. 280-282 (2018)] and Pilon v1.23 [Walker, B.J. et al. Pilon: An integrated tool for comprehensive microbial variant detection and genome assembly improvement. Plos One **9** (2014)] was carried out using paired-end (PE) Illumina 10X Linked-Reads sequencing reads (ca. 174X coverage). We obtained a

total of 102 contigs (total assembly size: 368.4Mb, N50: 11.3 Mb, L50: 12).

For Doublon 106X raw reads, PacBio and 6.8X (2.6 Gb) ONT reads were combined and the assembly was performed using canu v.1.6 [Koren, S. et al. Canu: scalable and accurate long-read assembly via adaptive k-mer weighting and repeat separation. *Genome Research* **27**, 722-736 (2017)] using genomeSize=380m, corOutCoverage=40 and minReadLength=1000 settings. We obtained 174 contigs (total assembly size: 372.3 Mb, N50: 11.1 Mb, 50: 12). We expected that ONT and PacBio sequencing would generate full sequences overlapping one or several contiguous *Vat*-related sequences. To check that feature, we mapped reads longer than the 0 kb reads used to construct the genome assemblies of Anso77 (37.9 Gb, 1.37 Mreads, N50: 33 kb, Max read length: 321 kb) and Doublon (34.7 Gb, 1.42 Mreads, N50: 27.7 kb, Max read length: 122 kb) using Minimap2 [32] on Anso77 and Doublon M5-M4 regions. Then we counted reads overlapping one, two and more contiguous *Vatx* for Anso77 and Doublon using the Integrative Genomics Viewer [40].
