# Supplementary material for: Building a cluster of NLR genes conferring resistance to pests and pathogens: the story of the Vat gene cluster in cucurbits
Source: Hortic Res. 2021 Apr 1;8:72. doi: 10.1038/s41438-021-00507-0 (PMC8012345; doi:10.1038/s41438-021-00507-0)
Supplement: Supplementary file 10 — Methods S2 Producing/predicting CDS from Vat homologs (C. melo) and Vat-related sequences (other cucurbits). For PI 161375, full cDNAs of Vat1, Vat2 and Vat-Rev were amplified by RT-PCR and sequenced. For other C. melo (Doublon, Anso77, Payzawat) and for C. sativus and C. lanatus only partial cDNAs spanning exon junctions were amplified and sequenced. [file 41438_2021_507_MOESM10_ESM.pdf]

***Producing/predicting CDS from Vat homologs (C. melo) and Vat-related sequences (other cucurbits)***

For all the different genotypes studied, total RNAs were isolated from young leaves using TRIZOL ®Reagent (THERMOFISHER, www.thermofisher.com) according to the manufacturer's instructions and measured up with Nanodrop (400 ng/µl). First-strand cDNAs were synthesized from 2 µg of total RNA using Super-script® III reverse transcriptase (INVITROGEN LIFE TECHNOLOGIES, www.thermofisher.com).

For PI 161375, *Vat1* and *Vat2* full cDNAs were amplified by long-range PCR with Z717F and R primer located in 5' and 3'-UTR (Table S2) using the Ex Taq Hot start enzyme (TAKARA BIO INC, Japan, www.takara-bio.com). Final RT-PCR products were purified after gel electrophoresis using the NucleoSpin Gel and PCR Clean-up kit (MACHEREY NAGEL, Germany, www.mn-net.com) and cloned with pGEM®-T Easy Vector Systems I (www.promega.com). Clones were screened by PCR with specific primers and plasmids were extracted with the QIAprep Spin Miniprep Kit (QIAGEN, www.qiagen.com). *VatRev* cDNA was directly amplified by RT-PCR using four specific primers located in 5' and 3'-UTR and in exon 1 (Table S2). For *Vat1, Vat2 and VatRev*, 5 to 6 primer pairs were used for sequencing full cDNAs by SANGER technology (GENOSCREEN, www.genoscreen.fr), and assemblies were conducted manually or with CAP3 (www.pbil.univ-lyon1.fr/cap3.php).

For Doublon, Anso77, Piel de Sapo T111 (for DHL92) and Payzawat, specific primers of each *Vatx* homolog (Table S2) were designed in the predicted exons. Partial cDNAs spanning all exon junctions, were amplified by RT_PCR and directly sequenced (without cloning) by SANGER technology.

For 16 other *C. melo* lines, we predicted CDSs from their genomic sequences by homology with CDS of *Vat1* from PI 161375. DNAs were isolated from young leaves using the DNeasy Plant Mini Kit according to the manufacturer's instructions. Long-Range PCRs were performed with common primers (Z717F and R) designed in 5' and 3'-UTR of the *Vat* homolog genes (Table S4). LR-PCR products (4.8 to 6.1 Kb) were purified after gel electrophoresis using the NucleoSpin Gel and PCR Clean-up Kit and cloned with pGEM®-T Easy Vector Systems I. Plasmids were extracted with the QIAprep Spin Miniprep Kit and sequenced by SANGER technology using a set of primer pairs distributed along the genes (Table S4); assemblies were performed manually or with CAP3.

For *C. sativus* cv 9930 and Gy14*,* specific primers of each *Vat*-related sequence (Table S2) were designed in the predicted exons. Partial cDNAs, spanning the junctions between the different exons, were amplified by RT_PCR and directly sequenced (without cloning) by SANGER technology. We did not get seeds from *C. lanatus* cv 97103, so we used *C. lanatus* cv Charleston Gray, that exhibited the same genomic sequence for the Vat-related region and we followed the strategy described above to validate the junctions between the different exons (Table S2).
