# Supplementary material for: Building a cluster of NLR genes conferring resistance to pests and pathogens: the story of the Vat gene cluster in cucurbits
Source: Hortic Res. 2021 Apr 1;8:72. doi: 10.1038/s41438-021-00507-0 (PMC8012345; doi:10.1038/s41438-021-00507-0)
Supplement: Supplementary file 11 — Methods S3 Automatic annotation of retrieved genomic areas. Structural annotation using MAKER v3.01.02 software and functional annotation using InterProScan and SwissProt databases. [file 41438_2021_507_MOESM11_ESM.pdf]

Relative frequency of nonsynonymous SNPs along *Vat*-related sequences

CDSs retrieved from *Vat*-related sequences in melon were aligned while considering three independent blocks - pre-LRR2/LLR2/post LRR2 - with the MUSCLE algorithm in the Seaview software package [39, 41] to investigate the relative frequency of nonsynonymous SNPs along *Vat*-related sequences.

Let X be a sequence of L codons observed on N accessions, where $c_{i,j}$ denotes the value of the codon at position i on line j, and $a_{i,j}$ denotes the value of the corresponding amino acid. Suppose that the N observed lines are an independent sample extracted from a family of lines A and $p(i)$ denotes the probability of nonsynonymous mutation that is assumed to be continuous along the sequence.

The probability $p(.)$ is estimated using nonparametric regression estimator [Hardle. Applied nonparametric regression (Cambridge University Press, 1990)]:

$$\hat{p}(i) = \frac{\sum_j \sum_{j'} \sum_{i'} w(x_i - x_{i'}) 1_{\{c_{i',j} \neq c_{i',j'}\}} 1_{\{a_{i',j} \neq a_{i',j'}\}}}{\sum_j \sum_{j'} \sum_{i'} w(x_i - x_{i'}) 1_{\{c_{i',j} \neq c_{i',j'}\}}}$$

where $w(.)$ is a Gaussian kernel with mean 0 and variance σ^2. An optimal variance σ^2 is estimated using a double kernel method which minimizes the integrated mean squared error $I = \sum_i E\left(\widehat{(p}(i) - p(i)\right)^2$).

A confidence band, under the assumption that p(.) is constant, is then built using a permutation method by randomly redistributing the $a_{i,j}$ values of each line j, then estimating a probability function $\tilde{p}(.)$, reiterating the procedure N times to get N estimations $\widetilde{p_k}(.)$ and computing a 0.95 confidence band as the 0.025 and 0.975 quantiles of the vector $(\widetilde{p_1}(i), .., \widetilde{p_N}(i))$ for each position i.
