# Supplementary material for: Building a cluster of NLR genes conferring resistance to pests and pathogens: the story of the Vat gene cluster in cucurbits
Source: Hortic Res. 2021 Apr 1;8:72. doi: 10.1038/s41438-021-00507-0 (PMC8012345; doi:10.1038/s41438-021-00507-0)
Supplement: Supplementary file 12 — Methods S4 Relative frequency of nonsynonymous SNPs along Vat-related sequences Probability of nonsynonymous mutation p(i), that is assumed to be continuous along the sequence and confidence band using an homemade permutation method. [file 41438_2021_507_MOESM12_ESM.docx]

**Relative frequency of nonsynonymous SNPs along *Vat*-related sequences**

CDSs retrieved from *Vat*-related sequences in melon were aligned while considering three independent blocks - pre-LRR2/LLR2/post LRR2 - with the MUSCLE algorithm in the Seaview software package [39, 41] to investigate the relative frequency of nonsynonymous SNPs along *Vat*-related sequences.

Let X be a sequence of L codons observed on N accessions, where $c_{i,j}$ denotes the value of the codon at position $i$ on line $j$, and $a_{i,j}$ denotes the value of the corresponding amino acid. Suppose that the N observed lines are an independent sample extracted from a family of lines *A* and $p(i)$ denotes the probability of nonsynonymous mutation that is assumed to be continuous along the sequence.

The probability $p(.)$ is estimated using nonparametric regression estimator [Hardle. Applied nonparametric regression (Cambridge University Press, 1990)]:

$$\hat{p}\left( i \right)= \frac{\sum_{j} \sum_{j'} \sum_{i'} w\left( x_{i}-x_{i^{'}} \right)1_{{\{c}_{i',j}\neq c_{i^{'},j^{'}}\}}1_{{\{a}_{i',j}\neq a_{i^{'},j^{'}}\}}}{\sum_{j} \sum_{j'} \sum_{i'} w\left( x_{i}-x_{i^{'}} \right)1_{{\{c}_{i',j}\neq c_{i^{'},j^{'}}\}}}$$

where $w(.)$ is a Gaussian kernel with mean 0 and variance $\sigma^{2}$. An optimal variance $\sigma^{2}$ is estimated using a double kernel method which minimizes the integrated mean squared error
 $I=\sum_{i} E\left( \hat{(p}\left( i \right)-p\left( i \right) \right)^{2})$.

A confidence band, under the assumption that p(.) is constant, is then built using a permutation method by randomly redistributing the $a_{i,j}$values of each line j, then estimating a probability function $\tilde{p}(.)$, reiterating the procedure N times to get N estimations $\tilde{p_{k}}\left( . \right)$ and computing a 0.95 confidence band as the 0.025 and 0.975 quantiles of the vector $\tilde{{(p}_{1}}\left( i \right),..,\tilde{p_{N}}\left( i \right))$ for each position $i$.
